# The Nrf2-HMOX1 pathway as a therapeutic target for reversing cisplatin resistance in non-small cell lung cancer via inhibiting ferroptosis

**DOI:** 10.1038/s41420-025-02564-z

**Published:** 2025-06-21

**Authors:** Ling Zuo, Xinru Zou, Jia Ge, Shuning Hu, Yixuan Fang, Yi Xu, Rui Chen, Sheng Xu, Guangyang Yu, Xiaorong Zhou, Lili Ji

**Affiliations:** 1 Department of Immunology, Medical School of Nantong University, Nantong, 226001 Jiangsu China; 2 Department of Pathology, Key Laboratory of Microenvironment and Translational Cancer Research, Medical School of Nantong University, Nantong, 226001 Jiangsu China; 3 Department of Pathology, the Second Affiliated Hospital of Soochow University, Suzhou, 215004 Jiangsu China; 4 Department of Pathology, Lianyungang Affiliated Hostital Of Nanjing University of Chinese Medicine, Lianyungang, 222004 Jiangsu China; 5 Department of Pathology, First Affiliated Hospital of Soochow University, Suzhou, 215006 Jiangsu China; 6 Jiangsu Province Key Laboratory in University for Inflammation and Molecular Drug Target, Nantong, 226001 Jiangsu China

**Keywords:** Non-small-cell lung cancer, Cell death, Chemotherapy

## Abstract

Cisplatin resistance is a major cause of poor prognosis in non-small cell lung cancer (NSCLC). Cisplatin-induced lung cancer cell death is associated with ferroptosis, a type of recently identified programmed cell death. Nrf2 is a critical component of the antioxidant system, and its protumorigenic activity in lung cancer has been extensively studied. However, the role of Nrf2 in cisplatin-induced ferroptosis and drug resistance remains elusive. Here, we demonstrated that cisplatin treatment induced ferroptosis in parental A549 lung adenocarcinoma cells and that this effect was significantly reduced in cisplatin-resistant A549/DDP cells. Knocking down Nrf2-sensitized A549/DDP cells to cisplatin-induced cytotoxicity by enhancing ferroptosis. Moreover, we demonstrated that Nrf2 promotes the expression of HMOX1 and that the Nrf2-HMOX1 pathway is critical for mediating its anti-ferroptotic function. Additionally, immunohistochemical analysis of NSCLC specimens revealed that Nrf2 expression was correlated with HMOX1 and high levels of Nrf2 and HMOX1 were associated with poor patient survival. These findings suggest that the HMOX1-Nrf2 pathway significantly influences treatment outcomes in NSCLC. Ultimately, we demonstrated that treatment with the Nrf2 inhibitor ML385 promoted ferroptosis by inhibiting the Nrf2-HMOX1 pathway, restoring cisplatin sensitivity in drug-resistant cells. Our findings provide insights into the mechanism underlying cisplatin resistance and suggest that targeting the Nrf2-HMOX1 pathway enhances cisplatin-induced ferroptosis and improves NSCLC treatment outcomes.

## Introduction

Lung cancer was the most frequently diagnosed cancer and the leading cause of cancer-related death worldwide in 2022 [1]. Non-small cell lung cancer (NSCLC) is the most common histological subtype of lung cancer [2]. The first-line treatment for patients with NSCLC is cisplatin (*cis*-diaminodichloroplatinum, CDDP or DDP)-based combination chemotherapy, which is also the standard treatment strategy for advanced NSCLC. Cisplatin induces tumor cell apoptosis by inducing DNA damage and the production of reactive oxygen species (ROS) and lipid reactive oxygen species (lipid ROS), leading to oxidative imbalance and cell death. However, the rapid development of resistance to cisplatin-based chemotherapy is a major contributor to poor prognosis. Therefore, a new strategy to overcome cisplatin resistance is urgently needed.

Ferroptosis induction has been reported as a potential strategy to reverse tumor drug resistance [3–6]. Ferroptosis is an iron-dependent form of nonapoptotic cell death caused by iron overload and the ROS-dependent accumulation of lipid peroxides and is characterized by distinctive ultrastructural changes, with mitochondrial alterations being the primary distinguishing feature [7]. Ferroptosis can be induced via extrinsic or intrinsic pathways. The extrinsic pathway involves glutathione depletion through the inhibition of cell membrane transporters such as the cystine/glutamate transporter system (SLC7A11/xCT) or iron overload by the activation of ferroportin, whereas the intrinsic pathway is activated by the inhibition of intracellular antioxidant enzymes such as glutathione peroxidase 4 (GPX4). According to studies on ferroptosis, acyl-CoA synthetase long-chain family member 4 (ACSL4) influences ferroptosis sensitivity by modulating lipid composition and may serve as a biomarker for ferroptosis [8, 9].

Lethal accumulation of lipid peroxides is a hallmark of ferroptosis. The defence mechanism that counters this process involves cellular antioxidant systems that directly neutralize lipid peroxides [10]. Nuclear factor E2-related factor 2 (Nrf2) is involved in the adaptive regulation of oxidative stress. Tumor cells effectively maintain the intracellular antioxidant state through Nrf2 activation as a result of mutations in genes such as *NRF2*, *KEAP1*, and *CUL3*, which accelerate tumor cell growth and contribute to the development of resistance to anti-tumor drugs [11, 12]. The Nrf2 pathway is abnormally activated in NSCLC, and high constitutive Nrf2 activity can promote malignant progression and drug resistance in lung cancer [13]. Many of the key components of anti-ferroptotic pathways are transcriptionally regulated by Nrf2, including GPX4, SLC7A11, iron metabolism genes (FLT/FTH1, SLC40A1) and ROS detoxification enzyme-related genes (*NQO1*, *GCLC*, *GCLM*) [14, 15]. Therefore, Nrf2 is a key regulator of ferroptosis. Clinical studies have demonstrated that the Nrf2 signaling pathway is a crucial defence mechanism against ferroptosis and is related to sorafenib resistance in liver cancer [16–18]. However, the downstream target genes of this antioxidant transcription factor that are involved in ferroptosis regulation have not been fully elucidated.

Heme oxygenase-1 (HMOX1) metabolizes heme into biliverdin/bilirubin, carbon monoxide, and ferrous iron. Biliverdin/bilirubin, a byproduct, has antioxidant activity and can exert cytoprotective effects against various stressors. Therefore, HMOX1 is commonly regarded as a survival molecule that plays an important role in cancer progression and can be directly upregulated by the activation of Nrf2 [19]. However, the role of HMOX1 in regulating ferroptosis is poorly understood. Here, we demonstrate that ferroptosis contributes to cisplatin-induced cell death and that Nrf2 suppresses this effect in cisplatin-resistant cells. Moreover, *HMOX1* is a key downstream target gene of Nrf2 signaling and is essential for Nrf2-mediated ferroptosis resistance. We further demonstrated that targeting the Nrf2-HMOX1 pathway, either genetically or pharmaceutically, restores the sensitivity of lung cancer cells to cisplatin. These findings suggest a potential therapeutic strategy to overcome cisplatin resistance in patients with lung cancer.

## Results

### Establishment of a cisplatin-resistant lung cancer cell model

**Fig. 1:**
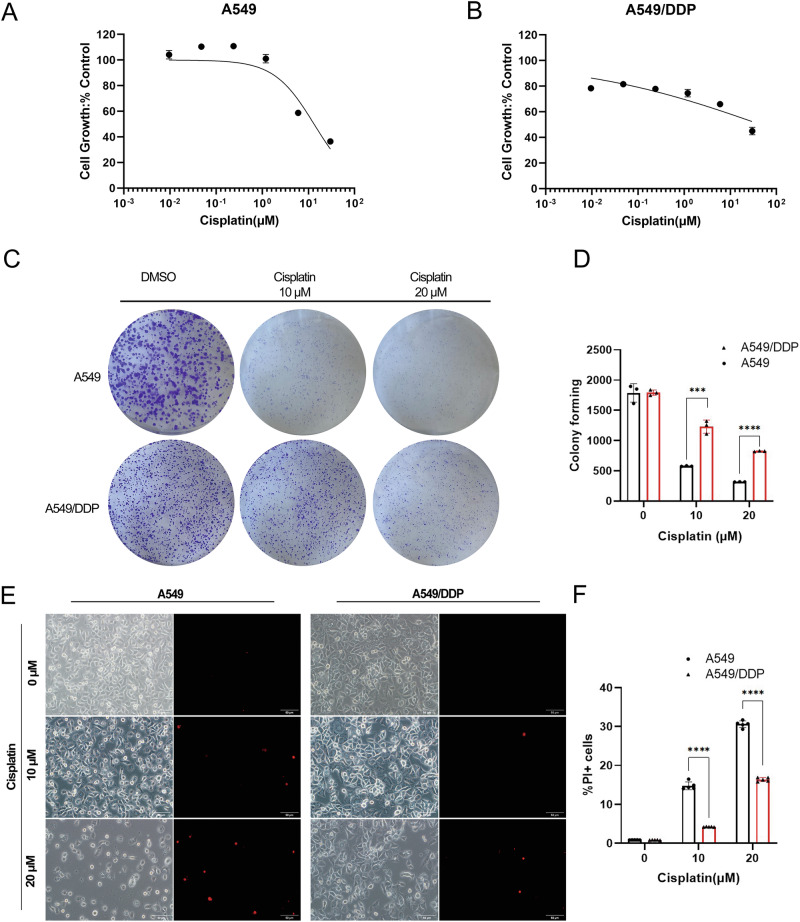
Cisplatin-induced death of lung cancer cells to varying degrees. **A**, **B** In vitro viability assay of parental A549 cells and cisplatin-resistant A549/DDP cells. A549 and A549/DDP cells in the logarithmic growth phase were seeded in 96-well plates and exposed to varying concentrations of cisplatin (0.0096 μM to 30 μM) for 48 h, after which cell viability was assessed. The data points represent averages of two replicates, with the curve showing the mean ± SEM. **C** In vitro clonogenic assay. A549 or A549/DDP cells were treated with DMSO or various concentrations of cisplatin for 48 h. After treatment, the cells were cultured for an additional 11–14 days without drugs, fixed with paraformaldehyde, and stained with crystal violet. Colonies were imaged via an inverted microscope. **D** The colonies were counted via ImageJ software. Colonies with more than 50 cells were scored via ImageJ. The experiments were conducted in triplicate (*n* = 3). **E**, **F** A549 and A549/DDP cells were treated with cisplatin for 48 h and then stained with propidium iodide (PI). Immunofluorescence and flow cytometry were performed to compare the degree of cell death following cisplatin treatment. The error bars represent the standard deviations of three replicates. Analysis was performed via unpaired *t-*tests, with ****p < 0.0001 relative to the control or differently treated groups.

The *KEAP1-*mutant human lung adenocarcinoma parental cell line (A549) and its derived cisplatin-resistant cell line (A549/DDP) were selected as in vitro cell models to study cisplatin resistance. A549/DDP cells were derived by treating parental A549 cells with gradually increasing doses of cisplatin in vitro. To confirm the establishment of the cisplatin-resistant cells, first, the half-maximal inhibitory concentration (IC_50_) and drug resistance index (RI) of the two cell lines were examined via a CCK-8 assay. The results of the CCK-8 assay revealed that the half-maximal inhibitory concentration (IC_50_) of cisplatin was 13.27 μM in A549 cells, whereas it was 45.94 μM in A549/DDP cells, with a drug resistance index (RI, the ratio of the IC_50_ between A549/DDP cells and A549 cells) of 3.46 (Fig. 1A, B). The colony formation assay results indicated that, compared with A549/DDP treatment, cisplatin treatment inhibited the in vitro clonogenicity of parental A549 cells more significantly (Fig. 1C, D). Immunofluorescence and flow cytometry results revealed that with increasing cisplatin concentration, the red fluorescence intensity of propidium iodide (PI) staining in both cell lines increased, with higher levels in A549 cells than in A549/DDP cells (Fig. 1E). Consistent with the immunofluorescence results, flow cytometry revealed a much greater percentage of PI-positive A549 cells than PI-positive A549/DDP cells (Fig. 1F). These results indicate the successful establishment of a cisplatin-resistant lung cancer cell model.

### Cisplatin triggers ferroptosis in lung cancer cells

**Fig. 2:**
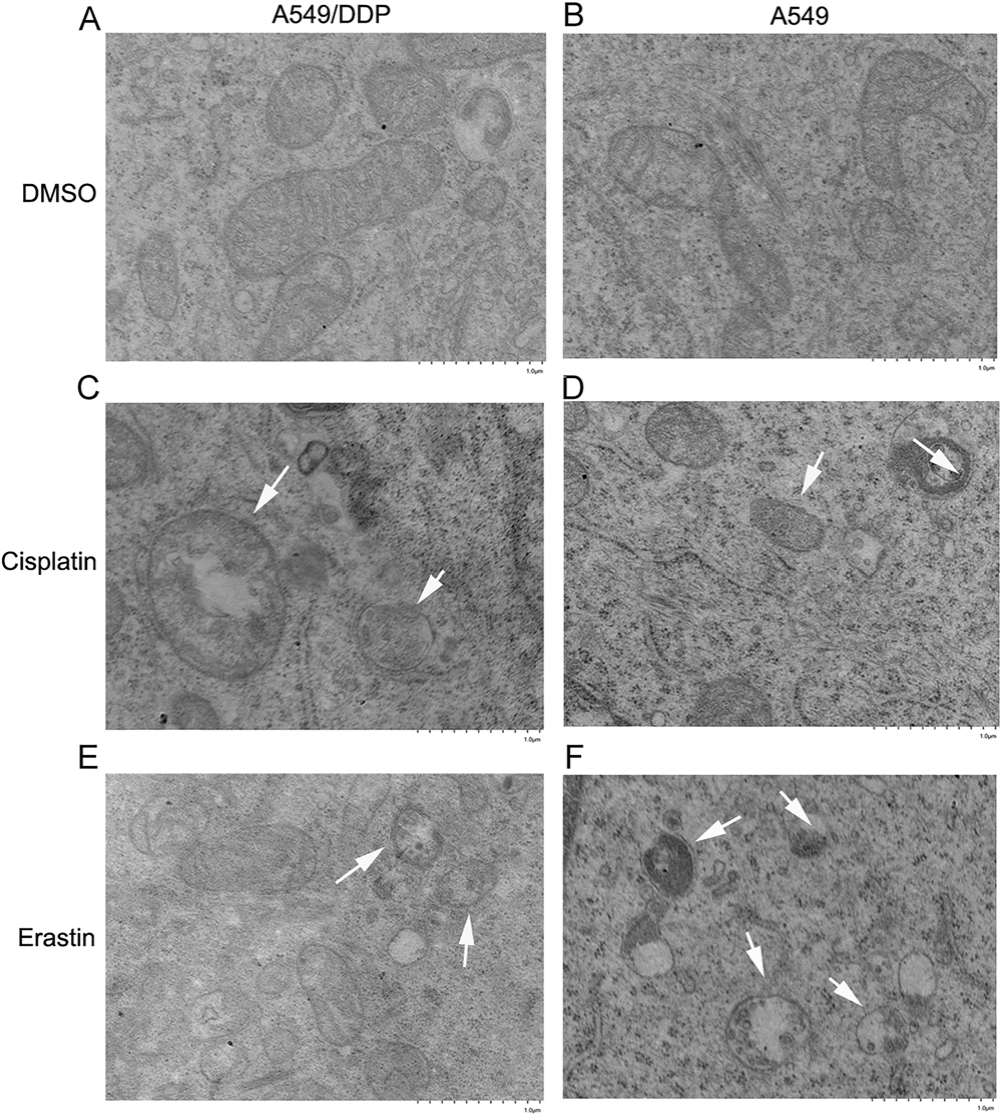
Transmission electron microscopy images of lung cancer cells, including A549/DDP (**A**, **C**, **E**) and A549 cells (**B**, **D**, **F**). The cells were treated with DMSO (**A**, **B**), the ferroptosis inducer erastin (10 μM), or cisplatin (20 μM) (**E**, **F**) for 48 h. The white arrowheads indicate alterations in mitochondrial ultrastructure, characterized by smaller mitochondria, less tubular morphology, and darker-stained membranes with distinct disruption of inner membrane folding. At least 10 cells were examined per treatment condition. Scale bar = 1 μm.

Compared with the DMSO group, which served as a negative control for ferroptosis (Fig. 2A, B), altered mitochondrial ultrastructures were observed in both cell lines following cisplatin treatment, including smaller mitochondria, less tubular morphology, and darker-stained membranes with distinct disrupted inner membrane folding (Fig. 2C, D), similar to the changes in the erastin-treated group, which served as a positive control for ferroptosis induction (Fig. 2E, F). We found that the changes in the mitochondrial ultrastructure were more pronounced in parental A549 cells than in A549/DDP cells.

**Fig. 3:**
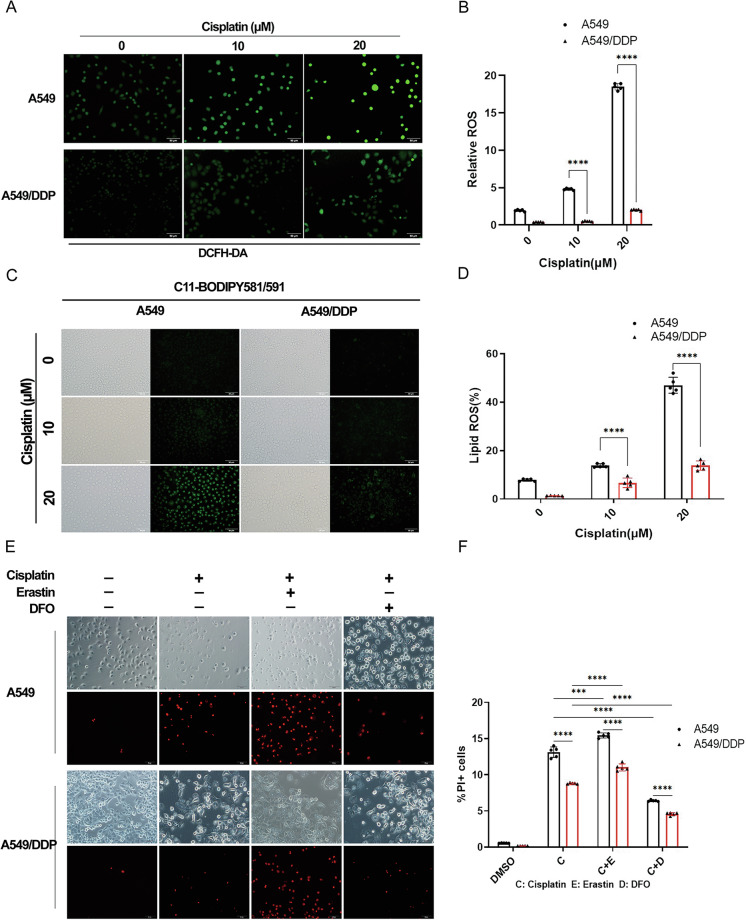
Measurement of the intracellular levels of ROS and lipid peroxidation and determination of cell death due to ferroptosis following cisplatin treatment. **A**–**D** A549 and A549/DDP cells in the logarithmic growth phase were seeded in 6-well plates and treated with cisplatin for 48 h. Subsequently, 10 μM DCFH-DA (**A**, **B**) or 2 μM C11-BODIPY581/591 (**C**, **D**) fluorescent probes were added to the medium in each well for 30 min to ensure coverage of the cell layer by the probe. Then, green fluorescence was observed via an immunofluorescence microscope (**A**–**C**), or the cells were collected to analyze the levels and differences in intracellular ROS and lipid peroxidation via flow cytometry (**B**–**D**). The error bars represent the standard deviation from three replicates. Analysis was performed via an unpaired *t*-test, with ****p < 0.0001 relative to the control or differently treated groups. **E** A549 and A549/DDP cells were seeded in 6-well plates, followed by stimulation with cisplatin (20 μM) alone or in combination with the ferroptosis inducer erastin (10 μM) or the iron chelator DFO (50 μM) for 48 h. Immunofluorescence staining with propidium iodide (PI) was performed to compare the differences in cell death levels after different treatments. **F** Flow cytometry was used to analyze the degree of cell death after different cisplatin treatments. The error bars represent the standard deviation from three replicates. Analysis was performed via an unpaired *t*-test, wi*t*h ****p < 0.0001 relative to the control or differently treated groups.

Immunofluorescence and flow cytometric analysis revealed that cisplatin increased the signal intensity of DCFH-DA (a probe for ROS production) in a dose-dependent manner, with a more pronounced increase in A549 cells than in A549/DDP cells (Fig. 3A, B). Consistent with these findings, the signal intensity of C11-BODIPY^581/591^ (probes for lipid peroxidation) was significantly greater in A549 cells than in control cells (Fig. 3C, D).

Consistent with the results shown in Fig. 1E, F, PI staining indicated that cisplatin treatment alone induced more cell death in A549 cells than in A549/DDP cells (Fig. 3E, F). Additionally, we found that while cotreatment with cisplatin and erastin (an inducer of ferroptosis) caused more cell death than did cisplatin alone in both cell lines, A549 cells were more sensitive to cotreatment than were A549/DDP cells (Fig. 3E). Although the addition of DFO (an iron chelator) suppressed cisplatin-induced cell death in both cell lines, DFO only partially reversed cisplatin-induced cell death. These findings suggest that although ferroptosis contributes significantly to cisplatin-induced cell death, other types of cell death, such as apoptosis, may also be involved in the cytotoxic activity of cisplatin (Fig. 3E, F).

### Cisplatin resistance in A549/DDP cells is associated with the defensive role of NRF2 in ferroptosis

**Fig. 4:**
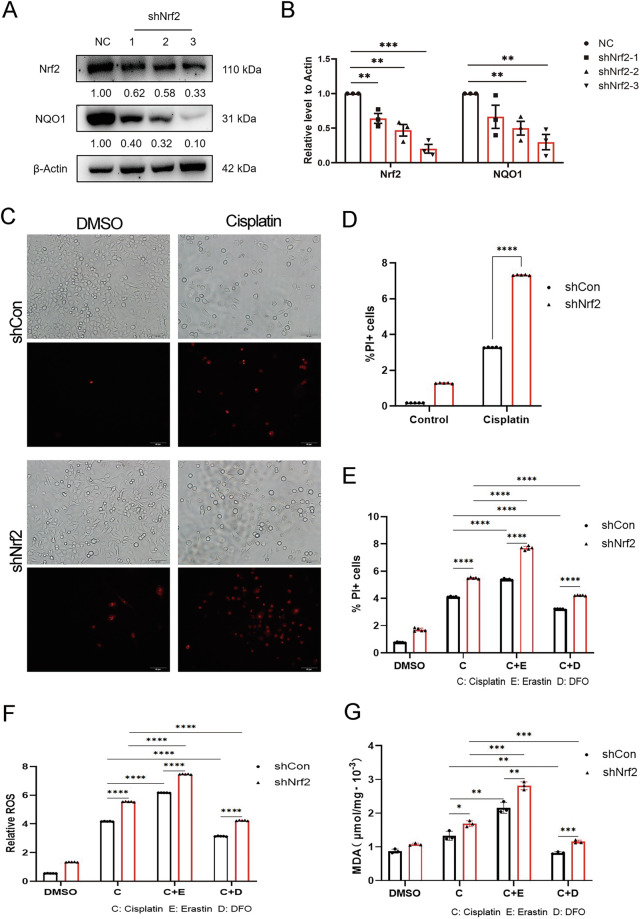
Cisplatin resistance in A549/DDP cells is associated with the protective role of Nrf2 against ferroptosis. **A**, **B** Western blotting was performed to determine the expression levels of Nrf2 and NQO1 in both NC- and Nrf2-shRNA-infected A549/DDP cells. The grayscale values of the protein bands, including those of Nrf2 and NQO1, were quantified relative to the β-actin loading control via ImageJ software. **C**, **D** A549/DDP cells were treated with 20 μM cisplatin for 48 h following Nrf2 knockdown. The number of PI-positive cells exposed to cisplatin was determined via fluorescence microscopy and flow cytometry. **E**–**G** A549/DDP cells were treated with cisplatin (20 μM) alone or with the ferroptosis inducer erastin (10 μM) or the iron chelator DFO (50 μM) for 48 h. **E** PI-positive cells exposed to cisplatin were stained and counted via flow cytometry. **F** The intracellular ROS level was assessed via an intracellular total ROS activity assay kit (orange fluorescence) following various treatments and analyzed via flow cytometry. **G** Changes in the intracellular lipid peroxidation levels were determined via an MDA detection kit and a microplate reader. The error bars represent the standard deviation from three replicates. Statistical analysis was performed via unpaired *t*-tests: **p < 0.01, ***p < 0.001, ****p < 0.0001 relative to the control or differently treated groups.

Considering the established function of NRF2 as a principal transcriptional regulator of antioxidant genes in response to oxidative stress [20, 21], along with its frequent hyperactivation in KEAP1-mutant lung adenocarcinoma [22, 23], we were prompted to investigate the role of NRF2 in mediating cisplatin resistance through the regulation of ferroptosis. Firstly, we constructed an Nrf2-knockdown A549/DDP cell line via shRNA lentivirus. After screening three different Nrf2 shRNA constructs, we found that shRNA3 was particularly effective in reducing the protein levels of Nrf2 and its target gene *NQO1* (Fig. 4A, B). Therefore, A549/DDP cells infected with the Nrf2-shRNA3 lentivirus (A549/DDP-Nrf2 shRNA) and control A549/DDP cells infected with lentivirus carrying nonspecific shRNA (A549/DDP-NC) were used for subsequent experiments. The immunofluorescence results indicated that the PI signal induced by cisplatin in A549/DDP cells was significantly enhanced following Nrf2 knockdown (Fig. 4C). Flow cytometric analysis accordingly demonstrated that cisplatin-induced cell death was significantly increased following Nrf2 knockdown (Fig. 4D). These results suggest that the resistance of A549/DDP cells to cisplatin is partly due to the activity of the Nrf2 signaling pathway.

Next, the cells were treated with cisplatin alone or cotreated with erastin or DFO following Nrf2 knockdown. Flow cytometric analysis revealed that consistent with the results shown in Fig. 4C, D, Nrf2 knockdown resulted in increased death in A549/DDP cells compared with control cells upon drug treatment. More importantly, the increase in cell death resulting from Nrf2 knockdown was further enhanced by the addition of erastin or reversed by cotreatment with DFO (Fig. 4E). Changes in the cellular ROS and lipid peroxidation levels after treatment with cisplatin alone or cotreatment with erastin or DFO followed a similar trend in these cell lines (Fig. 4F, G). These results suggest that Nrf2 knockdown could sensitize lung cancer cells to cisplatin through increased ferroptosis.

### The Nrf2-HMOX1 axis is involved in ferroptosis defence and cisplatin resistance

**Fig. 5:**
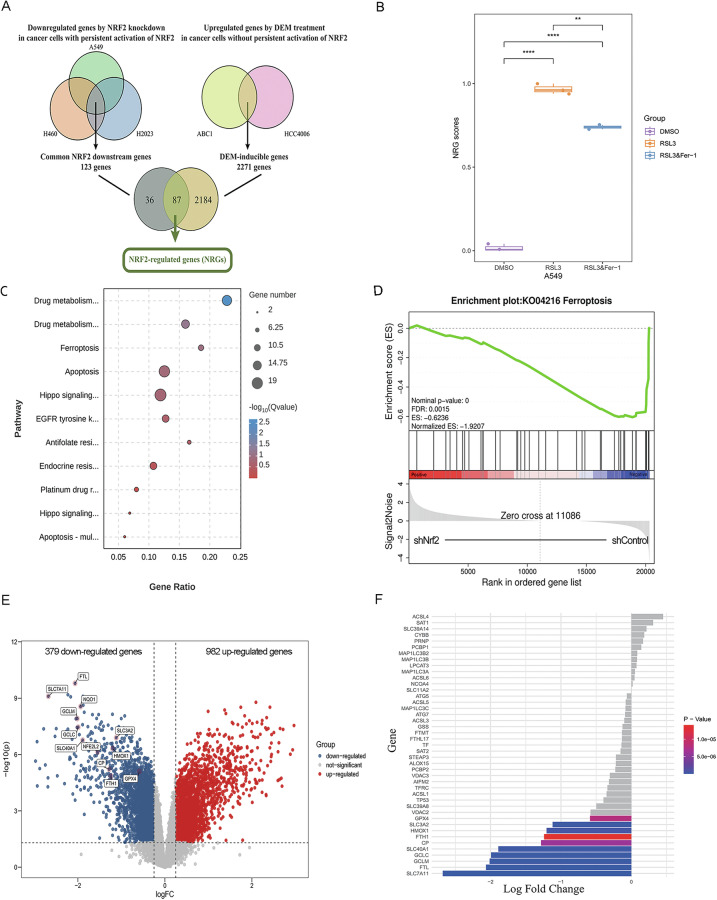
Profiling of Nrf2-regulated transcriptional networks and modulation of ferroptosis in lung cancer cells. **A** A conserved Nrf2 activity signature in lung cancer cells identified through a comprehensive multi-dataset analysis (GSE118841: Nrf2 knockdown; GSE118842: Nrf2 activation). **B** Single-sample Gene Set Enrichment Analysis (ssGSEA) demonstrating dynamic NRG scores across different treatment groups (DMSO, RSL3, and RSL3 combined with Fer-1) based on the dataset (GSE247883). **C**–**F** RNA sequencing analysis of A549/DDP cells subsequent to Nrf2 knockdown. **C** Bubble plot illustrating KEGG enrichment analysis of pathways significantly altered in the control and Nrf2-knockdown groups. **D** GSEA based on the KEGG database revealed significant differences in a set of genes related to ferroptosis pathways between the control and Nrf2-knockdown groups. **E** Volcano plot depicting genes that were differentially expressed between the control and Nrf2-knockdown groups. Genes with a false discovery rate (FDR) parameter below 0.05 and an absolute fold change ≥2 were deemed differentially expressed. **F** Bar chart created with the ggplot2 (v.3.5.1) package highlighting the top 10 downregulated genes relatated to ferroptosis following Nrf2 knockdown.

To further confirm the defensive role of Nrf2 in ferroptosis, we initially conducted a bioinformatic analysis of Nrf2-driven transcriptional networks. Through the integration of multiple datasets (GSE118841: Nrf2 knockdown; GSE118842: Nrf2 activation) [24], we identified 87 conserved Nrf2-regulated genes (NRGs), which collectively constitute a robust NRF2 activity signature in lung cancer cells (Fig. 5A). Subsequently, we explored the functional relationship between NRGs and ferroptosis. In the context of RSL3-induced ferroptosis (GSE247883) [25], single-sample Gene Set Enrichment Analysis (ssGSEA) revealed significantly elevated NRG scores, which were reversed by co-treatment with the ferroptosis inhibitor Fer-1 (Fig. 5B). These findings indicate that ferroptosis stress enhances Nrf2 signaling, serving as a compensatory survival mechanism for lung cancer cells.

Next, we conducted RNA-seq on two cell lines (A549/DDP and Nrf2-knockdown A549/DDP). The KEGG enrichment analysis bubble plot indicated significant differences in cell signaling pathways, including drug metabolism, ferroptosis, and apoptosis, between the two groups (Fig. 5C). The GSEA results revealed that the differentially expressed genes (DEGs) were enriched mainly in the ferroptosis pathway (Fig. 5D). Volcano plot analysis showed a total of 1361 DEGs between the control group and the Nrf2-knockdown group, which includes 982 upregulated and 379 downregulated genes (Fig. 5E). And it revealed that Nrf2 and multiple target genes, including those involved in glutamate/cystine transport (*SLC7A11*), iron metabolism (*FLT/FTH1*, *SLC40A1*), and ROS detoxification enzyme-related genes (*NQO1*, *GCLC*, *GCLM*), were downregulated after Nrf2 knockdown (Fig. 5E). These findings are consistent with previous findings concerning the involvement of Nrf2 target genes in ferroptosis, thus further verifying the accuracy of our RNA-seq results. Notably, among these genes, heme oxygenase 1 (*HMOX1*) was also significantly downregulated (Fig. 5E), and it was among the top 10 downregulated genes related to ferroptosis (Fig. 5F).

**Fig. 6:**
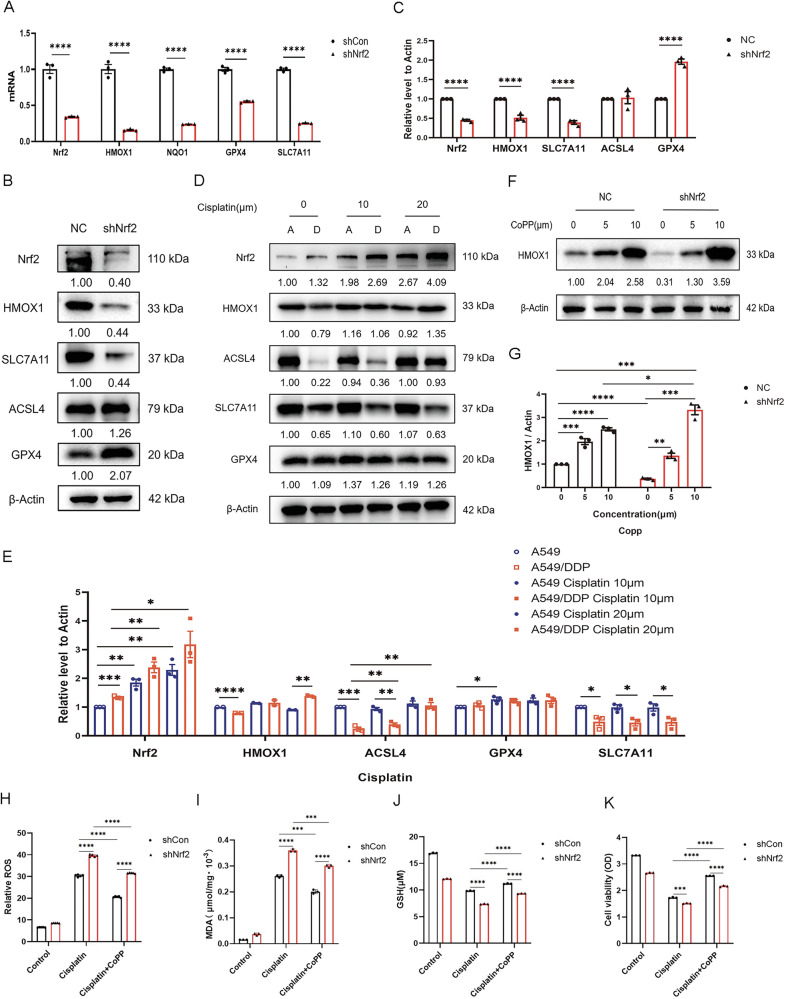
The Nrf2-HMOX1 axis is involved in defence against ferroptosis and cisplatin resistance. **A** Total RNA was extracted from A549/DDP cells following Nrf2 knockdown, and RT-PCR was performed to quantify the transcript levels of Nrf2 and its downstream target genes, including *HMOX1*. **B**, **C** Western blot analysis of Nrf2, HMOX1, SLC7A11, ACSL4, and GPX4 following Nrf2 knockdown in A549/DDP cells. **D**, **E** Western blot analysis of Nrf2, HMOX1, ACSL4, SLC7A11, and GPX4 in A549 (**A**) and A549/DDP (**D**) cells treated with various concentrations of cisplatin for 48 h. **F**, **G** A549/DDP cells transfected with negative control (NC) or Nrf2-shRNA were treated with CoPP, an inducer of HMOX1, for 48 h. Western blot analysis was conducted to assess HMOX1 expression levels in both cell lines. **H** Intracellular ROS levels were assessed via DCFH-DA (10 μM) following cisplatin treatment alone or cotreatment with the HMOX1 inducer CoPP and analyzed via flow cytometry. **I** Changes in the intracellular lipid peroxidation levels were analyzed with an MDA detection kit combined with a microplate reader. **J** The intracellular GSH level was measured via a GSH and GSSG assay kit. **K** Cell viability was assessed via a Cell Counting Kit following treatment with cisplatin alone or in combination with the HMOX1 inducer CoPP (10 μM). All WB experiments were repeated three times with similar results. The grayscale values of the protein bands were quantified relative to the β-actin loading control via ImageJ software. The error bars represent the standard deviations from three replicates. Statistical analysis was performed via unpaired *t*-tests, with *p < 0.05, **p < 0.01, ***p < 0.001, and ****p < 0.0001 indicating significance relative to the control or differently treated groups.

Subsequent RT-PCR and western blot analyses further verified the sequencing results (Fig. 6A–C), as HMOX1, along with SLC7A11, was reduced at both the mRNA and protein levels following Nrf2 knockdown (*p* < 0.0001), with slight upregulation of the ACSL4 protein (No statistically significant difference), which is a biomarker for ferroptosis [26]. Notably, Nrf2 knockdown significantly reduced GPX4 mRNA levels (*p* < 0.0001) but, paradoxically, increased its protein expression (*p* < 0.0001) (Fig. 6B, C). This discrepancy likely reflects compensatory mechanisms, such as feedback activation to mitigate Nrf2 depletion and post-transcriptional regulation of GPX4. Collectively, these findings suggest that *HMOX1* is a potential target gene downstream of the Nrf2 pathway involved in the regulation of ferroptosis.

Moreover, Western blot results revealed that the basal expression level of Nrf2 protein was significantly elevated in A549/DDP cells compared to parental A549 cells (*p* < 0.001), which indicated that Nrf2 may contribute to cisplatin reistance. Cisplatin treatment further increased its protein level in a dose-dependent manner in both A549 and A549/DDP cells, with higher expression in cisplatin-resistant A549/DDP cells (Fig. 6D, E). Consequently, the expression of HMOX1 was upregulated alongside that of Nrf2 following cisplatin treatment, and its level was much higher in A549/DDP cells than in A549 cells in the 20 µM group (*p* < 0.01) (Fig. 6D, E). Notably, compared with that in A549 cells, the basal expression of the ferroptosis-promoting gene ACSL4 in drug-resistant A549/DDP cells was significantly lower (*p* < 0.001), and its expression increased progressively with increasing cisplatin concentration. Although it increased significantly in the 20 µM group, its level was still lower than that in the A549 cell group (Fig. 6D, E). Western blot analysis (under nondenatured conditions) revealed that SLC7A11 baseline expression was lower in A549/DDP cells compared to A549 control cells (*p* < 0.05), while GPX4 levels remained comparable between both cell lines. Cisplatin treatment (20 µM) did not significantly alter SLC7A11 or GPX4 expression in either group (Fig. 6D, E). These findings suggest that the upregulation of the Nrf2/HMOX1 axis, combined with the downregulation of ACSL4, may play a role in the resistance to cisplatin-induced ferroptosis observed in A549/DDP cells.

To investigate the role of the Nrf2-HMOX1 axis in cisplatin-induced ferroptosis, we conducted rescue experiments following Nrf2 knockdown by utilizing cobalt protoporphyrin (CoPP), a well-established inducer of HMOX1 known for its protective effects against ischemia/reperfusion injury [27], and verified the changes in ferroptosis phenotype. The western blot results revealed that 10 μM CoPP significantly increased HMOX1 expression in control A549/DDP cells (*p* < 0.0001) and restored its expression following Nrf2 knockdown (*p* < 0.001), without inducing cytotoxicity (Fig. 6F, G), which is consistent with the RT-PCR results (Supplementary Fig. S1A). In contrast, initial evaluations of CP-312, a proposed HMOX1 agonist in cardiomyocytes [28] demonstrated cell line-specific effects. Specifically, CP-312 increased HMOX1 expression in PC-9 cells, yet paradoxically decreased HMOX1 expression in A549 cells and its derivatives at a concentration of 10 μM (Supplementary Fig. S1B, C). Higher concentrations (>10 μM) resulted in significant cytotoxicity, indicating that its utility for mechanistic studies in this context may be limited. Therefore, a 10 μM concentration of CoPP was used for subsequent experiments. Consistent with the results shown in Fig. 4F, the results of the intracellular ROS assay revealed that Nrf2 knockdown resulted in increased ROS levels in A549/DDP cells compared with those in control cells following cisplatin treatment. However, induction of HMOX1 with CoPP reduced the excessive ROS burden resulting from Nrf2 knockdown (Fig. 6H). Changes in MDA following treatment with cisplatin alone or preconditioning with CoPP showed a similar trend in these cell lines (Fig. 6I). GSH, a major cellular antioxidant, is highly reactive to lipid ROS. A decreased level of GSH is considered to be a marker of oxidative stress. As expected, Nrf2 knockdown resulted in decreased GSH levels in A549/DDP cells compared with those in shRNA control cells following cisplatin treatment. However, induction of HMOX1 with CoPP attenuated the additional depletion of GSH caused by Nrf2 knockdown (Fig. 6J). Accordingly, cell viability assays demonstrated that Nrf2 knockdown resulted in decreased cell viability due to oxidative stress induced by cisplatin. However, preconditioning cells with CoPP markedly alleviated the additional lethality caused by Nrf2 knockdown (Fig. 6K). These results suggest that the role of the Nrf2 pathway in preventing ferroptosis and promoting cisplatin resistance is HMOX1 dependent.

### Clinical relevance of HMOX1 and Nrf2 expression in NSCLC

**Fig. 7:**
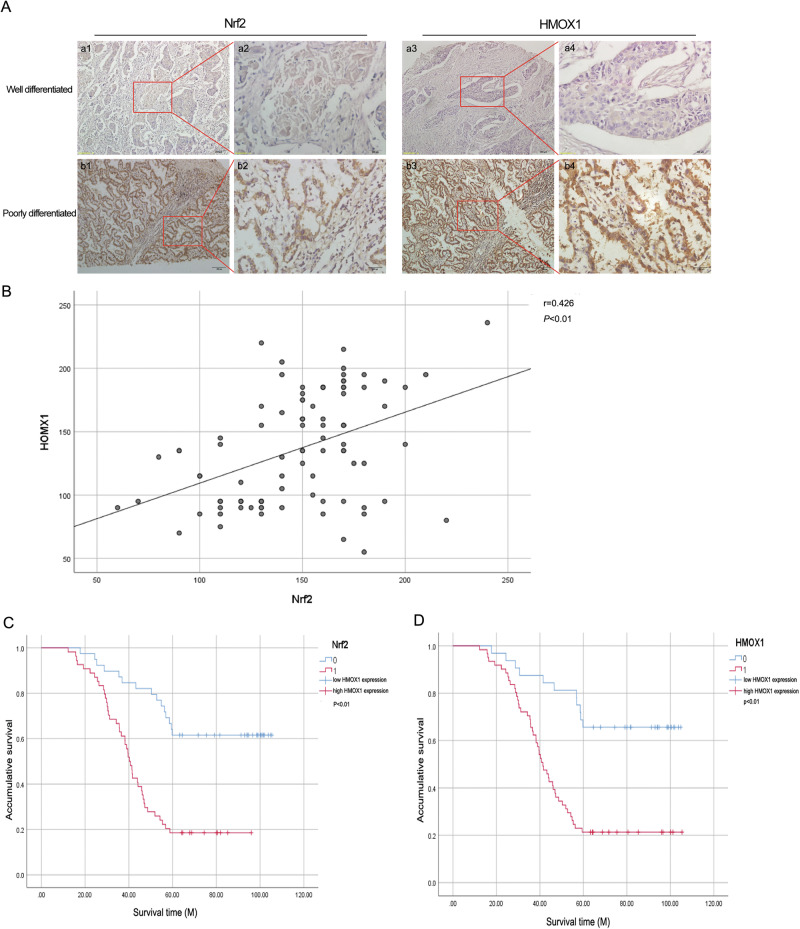
Clinical relevance of HMOX1 and Nrf2 expression in NSCLC. **A** Immunohistochemical staining of Nrf2 (a1-a2; b1-b2) and HMOX1 (a3-a4; b3-b4) in NSCLC specimens. Paraffin-embedded tissue sections were stained with antibodies against Nrf2 and HMOX1, followed by counterstaining with hematoxylin. Representative images depicting well-differentiated (a1-a4) and poorly differentiated tumor tissue (b1-b4) are shown (scale bar = 200 μm). **B** The relationship between HMOX1 and Nrf2 expression was assessed via the H score determined via IHC staining. The data were analyzed via the Social Science (SPSS) software package. The Pearson correlation test was used to determine the relationship, with significance set at *p* < 0.01. **C**, **D** Kaplan–Meier survival analysis based on the IHC expression status of Nrf2 and HMOX1. A log-rank (Mantel–Cox) test was conducted to assess the significance of differences.

We examined the expression of HMOX1 and Nrf2 in 93 treatment-naïve NSCLC specimens via immunohistochemistry. Representative images of Nrf2 and HMOX1 immunohistochemical staining are shown in Fig. 7A. As expected, HMOX1 was more highly expressed in poorly differentiated tumors than in well-differentiated tumors, which was consistent with Nrf2 expression. A Pearson correlation test was performed with the H score further to confirm the correlation between HMOX1 and Nrf2 expression. A significant positive correlation was found between the expression status of HMOX1 and that of Nrf2. As shown in Fig. 7B, the Pearson correlation coefficient (r) for HMOX1-Nrf2 was 0.426 (*p* < 0.01). In addition, we separated the samples into groups on the basis of low or high expression of Nrf2 and HMOX1 and evaluated their expression relative to each other as well as to clinicopathologic variables via Pearson’s *χ*^2^ test (Table 1). Consistently, a significant positive correlation was found between the expression of HMOX1 and that of Nrf2 (*p* = 0.0004). There was also a significant positive correlation between HMOX1 expression and clinical stage (*p* = 0.030), lymph node metastasis (*p* = 0.003), and distant metastasis status (*p* = 0.003). Overall, these data indicate that compared with low HMOX1 expression, high HMOX1 expression is associated with increased disease stage, lymph node metastasis, and distant metastasis. To further characterize the relationships between clinicopathological variables, including HMOX1 and Nrf2 expression, and clinical prognosis, the Pearson *χ*^2^ test was performed, as shown in Supplementary Table S1. Univariate survival analysis revealed that HMOX1 expression (*p* < 0.0001), clinical stage (*p* = 0.003), pathological grade (*p* = 0.047), lymph node metastasis (*p* < 0.0001), and distant metastasis status (*p* = 0.004) were prognostic indicators of overall survival. In addition, Kaplan–Meier analysis revealed that high expression of HMOX1 and Nrf2 was associated with poor overall survival in patients (Fig. 7C, D, *p* < 0.01). These findings suggest that HMOX1 expression in the NSCLC cohort is correlated with Nrf2 expression and contributes to the development of malignant properties in NSCLC.

**Table 1:** Clinicopathological features of NSCLC in relation to HMOX1 expression.

Clinicopathological parameters	Total	HMOX1		*p* value
		Low (*N* = 43)	High (*N* = 50)	
Gender				
Male	57	27 (48.21%)	29 (51.79%)	0.638
Female	36	16 (43.24%)	21 (56.76%)	
Age (years)				
<60	44	22 (51.16%)	21 (48.84%)	0.377
>60	49	21 (42.00%)	29 (58.00%)	
Smoking status				
Yes	46	20 (44.44%)	25 (55.56%)	0.737
No	47	23 (47.92%)	25 (52.08%)	
Tumor size (cm)				
<3	54	28 (51.85%)	26 (48.15%)	0.201
≥3	39	15 (38.46%)	24 (61.54%)	
Clinical stage (TNM)				
I + II	64	31 (55.36%)	25 (44.64%)	0.030^*^
III + IV	29	12 (32.43%)	25 (67.57%)	
Pathology grade				
Poor	21	6 (24.00%)	19 (76.00%)	
Moderate	40	17 (54.84%)	14 (45.16%)	0.371
Well	32	20 (54.05%)	17 (45.95%)	
Lymph node metastasis				
No	40	20 (68.97%)	9 (31.03%)	0.003^**^
Yes	53	23 (35.94%)	41 (64.06%)	
Distant metastasis status				
M0	81	39 (54.93%)	32 (45.07%)	0.003^**^
M1	12	4 (18.18%)	18 (81.82%)	
Nrf2 expression				
Low	38	26 (68.42%)	12 (31.58%)	0.0004^***^
High	55	17 (30.91%)	38 (69.09%)	

Statistical analyses were performed by Pearson χ2 test. The details of how expression was classified into high and low groups are described in “Materials and methods”.

*p < 0.05; **p < 0.01; ***p < 0.001.

### ML385 promoted ferroptosis and improved the sensitivity of lung cancer cells to cisplatin by inhibiting the Nrf2-HMOX1 pathway

**Fig. 8:**
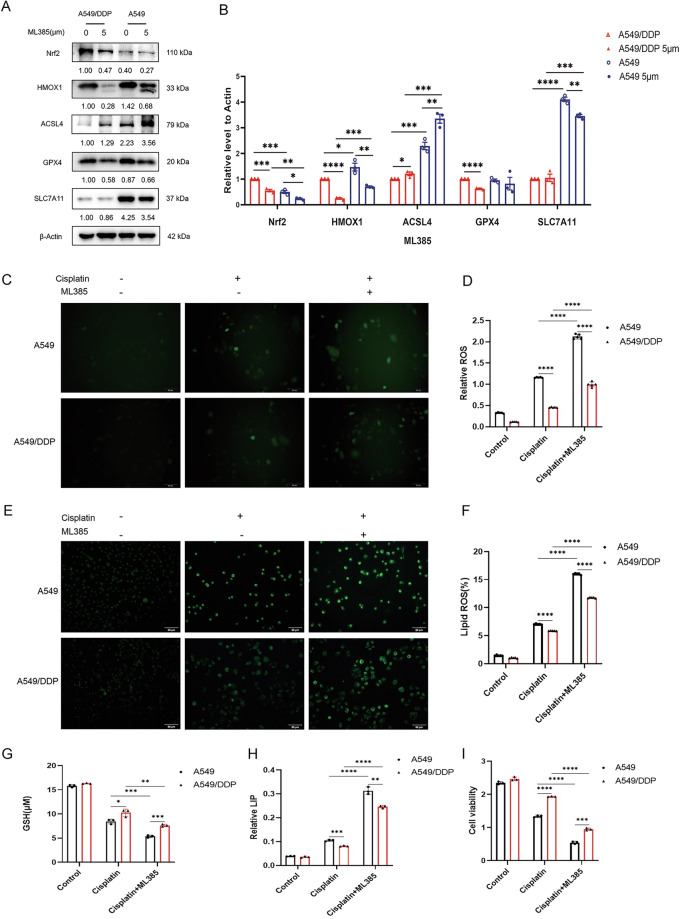
ML385 promotes ferroptosis by inhibiting the Nrf2-HMOX1 pathway and enhances the sensitivity of lung cancer cells to cisplatin. **A**, **B** Both A549 and A549/DDP cells were treated with the Nrf2 inhibitor ML385 (5 μM) for 48 h. Western blot analysis was conducted to assess the expression levels of Nrf2, HMOX1, ACSL4, GPX4, and SLC7A11 in both cell lines. The WB experiment was repeated three times with similar results. The grayscale values of the protein bands were quantified relative to the β-actin loading control via ImageJ software. **C**, **D** Both A549 and A549/DDP cells were treated with cisplatin (20 μM) alone or in combination with ML385 (5 μM). The intracellular ROS level was assessed via DCFH-DA (10 μM) and analyzed via flow cytometry. **E**, **F** Intracellular Lip ROS levels were assessed via C11-BODIPY581/591 (2 μM) and analyzed via flow cytometry. **G** Intracellular GSH levels were measured via GSH and GSSG assay kits. **H** A LIP assay was performed to quantify labile iron levels. **I** Cell viability was assessed via a Cell Counting Kit. Statistical analysis was performed via unpaired *t-*tests, with significance levels denoted as **p < 0.01, ***p < 0.001, and ****p < 0.0001 relative to the control or differently treated groups.

Western blot assays revealed that 5 μM ML385 (an Nrf2-specific inhibitor) inhibited the expression of Nrf2 in A549/DDP (*p* < 0.001), concomitant with significant downregulation of HMOX1 (*p* < 0.0001), GPX4 (*p* < 0.0001)and upregulation of ACSL4 (*p* < 0.05), while the level of SLC7A11 remained largely unchanged following ML385 treatment (Fig. 8A, B). Accordingly, real-time PCR revealed that ML385 treatment in A549/DDP significantly reduced the mRNA expression of Nrf2 and HMOX1 (*p* < 0.01) (Supplementary Fig. S1D). Immunofluorescence and flow cytometry assays revealed that the addition of cisplatin resulted in increased intracellular ROS and lipid peroxidation levels in both A549 and A549/DDP cells, with the increase being more obvious in the cisplatin-sensitive A549 cells, which is consistent with the results shown in Fig. 3. Moreover, ML385 combined with cisplatin further increased the levels of ROS and lipid peroxidation (Fig. 8C–F). Conversely, the GSH-GSSG assay revealed that changes in the GSH level followed the opposite trend, indicating an increase in oxidative stress (Fig. 8G).

The labile iron pool (LIP) assay revealed that consistent with the changes in ROS and lipid ROS levels, the increase in LIP was more robust in the ML385 plus cisplatin treatment group than in the group treated with cisplatin alone (Fig. 8H). Therefore, both A549 and A549/DDP cell viability decreased most significantly in the ML385 plus cisplatin treatment group (Fig. 8I). These results suggest that the inhibition of Nrf2-HMOX1 through the ML385 drug combination aggravates the cisplatin-induced increase in the ROS burden and ferroptosis, thereby restoring the cisplatin sensitivity of cisplatin-resistant lung cancer cell lines.

## Discussion

We demonstrated that cisplatin-based chemotherapy induces ferroptosis in lung cancer cells and that cisplatin resistance is correlated with reduced ferroptosis. Nrf2 plays an important role in mitigating lipid peroxidation and ferroptosis by activating cytoprotective genes involved in iron metabolism, GSH metabolism, and ROS detoxification [15]. In a previous study, we reported that lung cancer exhibited the highest frequency of alterations among the 31 surveyed malignancies [29]. The high frequency of Nrf2-Keap1 mutations enhances the ability of tumors to resist oxidative stress damage, increasing their resistance to ferroptosis and chemotherapy. However, it remains to be determined whether the resistance of lung cancer cells to cisplatin is linked to the role of Nrf2 in preventing ferroptosis. Our study suggested that the resistance of A549/DDP cells to cisplatin is linked to Nrf2 signaling activity and that Nrf2 knockdown sensitized lung cancer cells to cisplatin through increased ferroptosis. Therefore, targeting Nrf2 to modulate lipid peroxidation and ferroptosis represents a viable therapeutic strategy, as discussed below.

HMOX1 plays a cytoprotective role against increased oxidative stress caused by chemotherapeutic agents, which promotes cell proliferation and metastasis [30, 31]. However, the regulatory role of HMOX1 in ferroptosis remains somewhat controversial, given that it acts as a dual regulator of iron and ROS homeostasis. Both biliverdin and bilirubin inhibit the peroxidation of lipids and proteins by scavenging ROS, suggesting that HMOX1 plays a role in cytoprotective defence mechanisms against ferroptosis [32–34]. However, other studies have indicated that HMOX1 induces ferroptosis through iron accumulation or other unknown mechanisms [35–37]. Moreover, HMOX1 activation also increases the expression of ferritin, which binds to ferrous iron and mitigates its pro-oxidant effects [38]. On the basis of these contradictory results, HMOX1 may play dual roles in ferroptosis, encompassing both protective and detrimental functions in different contexts. The mechanism of its involvement in the regulation of ferroptosis, particularly its role in cancer chemotherapy, remains elusive and warrants further exploration. We showed that targeting the Nrf2-HMOX1 axis to mediate ferroptosis might have therapeutic utility, particularly in the context of chemotherapy in cancers. However, the precise underlying mechanism requires further investigation.

Given the abnormal activation and carcinogenic role of Nrf2 in a variety of tumors, the development of effective Nrf2-specific inhibitors and their clinical application has become a research hotspot in recent years. Our study results suggest that drugs targeting Nrf2-HMOX1 might serve as a viable approach for utilizing ferroptosis to kill resistant lung cancer cells via a specific inhibitor of Nrf2. We found that ML385 aggravated the cisplatin-induced increase in ROS burden and ferroptosis by inhibiting the Nrf2-HMOX1 axis, restoring the sensitivity of resistant lung cancer cell lines to cisplatin. Given that the poor prognosis of patients with lung cancer is often due to the rapid development of resistance to cisplatin-based chemotherapy, our findings could represent a promising strategy for NSCLC treatment.

## Materials and methods

### Cell culture and reagents

The NSCLC cisplatin-resistant cell line A549/DDP (CL-0519) and its parental cell line A549 (CL-0016) were purchased from Procell Life Science & Technology Co., Ltd. The cells were cultured in RPMI-1640 medium supplemented with 10% fetal bovine serum, 5 mg/mL penicillin-streptomycin, and 1–2 µg/ml DDP specific for A549/DDP cells at 37 °C under 5% CO_2_. The drug-resistant cell line (A549/DDP) was derived from the parental A549 cell line by gradually increasing the cisplatin dose in vitro.

### Cell viability, clonogenic, and cell death assays

Cell viability was measured via a Cell Counting Kit-8 (c0042; Beyotime, China) according to the manufacturer’s instructions. To assess clonogenic survival following drug exposure, the cells were cultured in a complete growth medium at 37 °C for 11–14 days and stained with crystal violet (C0121-100 ml, Beyotime, China) solution. Cell death following different treatments was measured via propidium iodide (PI, P4170-25MG; Sigma-Aldrich, USA) staining with a flow cytometer (BD Biosciences, USA).

### Transmission electron microscopy

A549 and A549/DDP cells were fixed with precooled 2.5% glutaraldehyde and then postfixed with 1% osmium tetraoxide solution. After stepwise dehydration in increasing concentrations of ethanol, the cells were embedded in Epon 812 epoxy resin (Sigma) and sectioned. Ultrathin sections were stained with lead citrate and uranyl acetate and imaged via transmission electron microscopy (JEOL Ltd., Tokyo, Japan).

### Determination of intracellular ROS production and lipid peroxidation

A549/DDP and A549 cells were seeded into 6-well plates at a density of 2 × 10^6^ cells per well. The next day, the cells were incubated in 2 mL of media supplemented with various drugs for 48 h. Then, the culture media was replaced with serum-free media containing either 10 μM DCFH-DA (S0033S, Beyotime, China) for cytosolic ROS or 2 μM C11-BODIPY581/591 (D3861, Thermo Fisher Science, USA) for lipid peroxidation. The cells were subsequently placed in the dark for 30 min, with gentle shaking every 5 min. The DCFH-DA-stained green fluorescence signal or the C11-BODIPY581/591-stained red fluorescence signal was observed under a fluorescence microscope (OLYMPUS, Japan). The shRNA vector GV493, containing a coding sequence for EGFP, was utilized to achieve Nrf2 knockdown in A549/DDP cells. To assess the cytosolic ROS levels in these Nrf2-deficient A549/DDP cells, a ROS 570 assay solution with orange fluorescence (KA4075, Abnova, China) was used. The cells were then incubated in a 5% CO_2_ environment at 37 °C for 1 h. The fluorescence signals were monitored at Ex/Em = 540/570 nm (cut off = 550 nm). The fluorescence was subsequently determined via flow cytometry (BD Biosciences, San Jose, CA). Each sample met the acquisition criteria of 10,000 events, and the results were analyzed with FlowJo v7.6 software.

### Immunohistochemical staining and analysis

The authorization for the use of fresh human tissue samples, tissue sections, and associated patient clinical data for this project was granted by the Ethics Committee of the Affiliated Hospital of Nantong University, Jiangsu Province, China (number: 2024–21). Written informed consent was obtained from all the patients, and patient specimens were collected in accordance with the Declaration of Helsinki 2013. Patient samples were obtained immediately after surgical resection and preserved at −80 °C. Immunohistochemical staining was performed as previously described [29]. The primary antibodies used were as follows: (1) Nrf2 (1:100, ab76026, Abcam, Inc.), (2) HMOX1 (1:50, sc-136960, Santa Cruz Biotechnology, Inc.). The staining intensity was coded as follows: 0 (negatively stained), 1 (poorly stained), 2 (moderately stained), and 3 (strongly stained). The staining intensity (0–3) was multiplied by the percentage of positively stained cells to calculate the H-scores, which ranged from 0 to 300.

### Statistical analysis

All the assays were conducted 3 times. The statistical significance of differences between treatment groups was evaluated via an unpaired *t*-test with GraphPad Prism 9 software. Differences were deemed statistically significant at a *P*-value of <0.05.

For more materials and methods, please refer to the Supplementary Materials.

### Supplementary information

Supplementary Fig S1. RT-PCR and WB results of gene expression following CoPP, CP-312 or ML385 treatments. Supplementary Table S1. Univariate analysis of clinicopathological parameters in NSCLC patients with respect to survival. Word file of supplementary material for material and methods. Word file of supplementary material for original western blots.

## Supplementary information

Supplementary material for material and methods

supplementary material for Fig S1

Supplementary material for Table S1

Supplementary material for original western blots

## Data Availability

RNA sequencing data for Nrf2-knockdown A549/DDP cells, as well as the corresponding control cells, are available in the Gene Expression Omnibus (GEO) database under the accession number GSE288129. The full code used during the current study is available at https://github.com/sangmm12/Nrf2.
