# Supplementary material for: The Nrf2-HMOX1 pathway as a therapeutic target for reversing cisplatin resistance in non-small cell lung cancer via inhibiting ferroptosis
Source: Cell Death Discov. 2025 Jun 21;11:287. doi: 10.1038/s41420-025-02564-z (PMC12182566; doi:10.1038/s41420-025-02564-z)
Supplement: Supplementary file 1 — Supplementary material for material and methods [file 41420_2025_2564_MOESM1_ESM.docx]

**2.7 Nrf2 knockdown in A549/DDP cells**

Nrf2 shRNA lentiviral constructs were purchased from Genechem Co, Shanghai, China. The targeting sequences for *NRF2* were as follows: GCTCCTACTGTGATGTGAAAT for shRNA1 (106660-1), GCTCAGTCACCTGAAACTTCT for shRNA2 (106661-1), and GCCCATTGATGTTTCTGATCT for shRNA3 (106662-2). The non-targeting shLuc TTCTCCGAACGTGTCACGT (CON313) was used as a negative control. The *NRF2*-shRNA or non-targeting shLuc were cloned into the lentiviral vector GV493 (hU6-MCS-CBh-gcGFP-IRES-puromycin; Shanghai Genechem Company, China). Subsequently, the *NRF2*-shRNA plasmids and negative control plasmid, combined with standard helper packaging plasmids, were transfected into 293T cells to produce lentiviruses using HiTrans G P. transfection regent (Genechem Co, Shanghai, China). The viral supernatants were applied to A549/DDP cells and, after 48 h, cells were subjected to puromycin (2 μg/mL, Beyotime, China) selection for 72 h.

**2.8 Western blotting**

Cells were cultured to reach 70% confluence before being treated with the specified drugs. The cells were lysed using a cell lysis buffer (P0013B, Beyotime, China) at 4˚C. The protein content in the supernatant was quantified using the BCA method (cat.P0012S, Beyotime, China). All protein samples were added with SDS-PAGE loading buffer before electrophoresis. For SLC7A11 protein samples (SLC7A11, Abcam, ab307601), they were placed at room temperature for 10 minutes and then added directly to the gel lane. The remaining protein samples needed to be heated in a 95°C metal bath for 10 minutes. After heating, they were centrifuged for 5 seconds and added to the gel lane after the samples returned to room temperature. A total of 10 μg protein was resolved by SDS-PAGE (P0012A, Beyotime, China) on 12% gels and then transferred to PVDF membranes. The membranes were treated with blocking buffer for 2 h and probed with primary antibodies at 4℃ overnight. After being washed three times in TBST for about 10 min each, the membranes was incubated with secondary antibodies at 37˚C for 1 h. The following primary antibodies were used: Nrf2 (ab137550, Abcam, USA; 16396-1-AP, Proteintech, China), HMOX1 (10701-1-AP, Proteintech, China), SLC7A11 (ab307601, Abcam, USA), NQO1 (sc-271116, Santa Cruz, USA), ACSL4 (22401-1-AP, Proteintech, China), GPX4 (30388-1-AP, Proteintech, China), goat anti-mouse IgG-HRP (abs20039, Absin, China), goat anti-rabbit IgG-HRP (abs20040, Absin, China), and β-actin (HRP-66009, Proteintech, China). All antibodies were diluted to between 1:1000 and 1:5000.

**2.9 Determination of MDA level assay**

Cell lysis buffer (150 μl; P0013, Beyotime, China) was added to the cells followed by incubation on ice for 10 min. A cell scraper was used to detach the cells, and the cell-buffer mixture was transferred to a 1.5 mL tube on ice. The tubes were vortexed every 10 min, a process that was repeated three times. The supernatant lysis buffer from each tube was collected by centrifugation at 12,000 g, 4˚C for 10 minutes. A 100 μl lysis sample was mixed with 200 μl of malondialdehyde solution (S0131, Beyotime, China) and incubated in the dark at 100℃ for 15 min. After all mixtures had cooled down to room temperature, they were centrifuged at 1000 g, 25˚C for 10 minutes. Next, 200 μl of supernatant from each tube was transferred to a 96-well plate and the absorbance was immediately measured at an OD of 532 nm. Additionally, a standard curve was constructed in accordance with the manufacturer’s instructions.

**2.10 RNA extraction and real-time PCR (RT-PCR)**

Cells were collected and RNA was isolated using the RNA-easy^TM^ Isolation Reagent (R701, Vazyme, China). The RNA concentration was assessed using a spectrophotometer (Thermo Fisher Scientific, USA). Subsequently, 1000 ng of total RNA per sample was utilized for a reverse-transcription reaction using the HiScript II Q RT SuperMix for qPCR(+g DNA wiper) (R223, Vazyme, China). Quantitative PCR was carried out on triplicate samples using the ChamQ Universal SYBR qPCR Master Mix (Q711, Vazyme, China), and the relative expression of the target gene under experimental and control conditions was calculated using the 2-^∆∆CT^ method. The primers for RT-PCR of the following genes were designed using Primer Bank. *NRF2*: upstream sequence: 5′-ATAGCTGAGCCCAGTATC-3′, downstream sequence: 5′-CATGCACGTGAGTGCTCT-3′. *HMOX1*: upstream sequence: 5′-AAGACTGCGTTCCTGCTCAAC-3′, downstream sequence: 5′-AAAGCCCTACAGCAACTGTCG-3′. *NQO1*: upstream sequence: 5′-TTGGAGTCCCTGCCATTCTGA-3′, downstream sequence: 5′-GGATCCCTTGCAGAGAGTACA-3′. *SLC7A11*: upstream sequence: 5′- TCCTGCTTTGGCTCCATGAACG-3′, downstream sequence: 5′- AGAGGAGTGTGCTTGCGGACAT-3′. *GPX4*: upstream sequence: 5′- ACAAGAACGGCTGCGTGGTGAA-3′, downstream sequence: 5′- GCCACACACTTGTGGAGCTAGA-3′. *ACSL4*: upstream sequence: 5′- GCTATCTCCTCAGACACACCGA-3′, downstream sequence: 5′- AGGTGCTCCAACTCTGCCAGTA-3′.

**2.11 Bioinformatic analysis of Nrf2-regulated transcriptional networks and ferroptosis modulation in lung cancer cells**

The transcriptomic datasets GSE118841, which involves NRF2 siRNA knockdown in A549, H460, and H2030 cell lines compared to non-targeting siRNA controls, and GSE118842, which examines NRF2 activation by DEM in ABC1 and HCC4006 cells [1], were integrated to identify conserved NRF2-regulated genes (NRGs). Benjamini-Hochberg (BH) correction-adjusted p-values were computed. Gene expression changes, whether decreases or increases, were deemed statistically significant if the adjusted p-values were below 0.05. An intersection analysis of genes responsive to NRF2 perturbation—specifically those downregulated following knockdown and upregulated following activation—was conducted to elucidate the NRGs. The activity of these NRGs was quantified using single-sample Gene Set Enrichment Analysis (ssGSEA) via the GSVA package[2]. Subsequently, the lung cancer dataset GSE247883, which involves RSL3 treatment with or without Fer-1 [3], was analyzed to calculate NRG scores. Statistical comparisons and visualizations were executed using the ggpubr package (<https://github.com/kassambara/ggpubr>) , and the data were normalized within the range of 0 to 1.

**2.12 RNA extraction, library construction, and transcriptome sequencing**

Total RNA was isolated using the Trizol reagent kit (15596026, Invitrogen, Carlsbad, CA, USA) in accordance with the manufacturer’s protocol. The quality of the RNA was evaluated using an Agilent 2100 Bioanalyzer (Agilent Technologies, Palo Alto, CA, USA) and verified by RNase free agarose gel electrophoresis. Following the isolation of total RNA, eukaryotic mRNA was enriched using Oligo(dT) beads. Then the enriched mRNA was fragmented into short fragments using fragmentation buffer and reverse- transcribed into cDNA by using NEBNext Ultra RNA Library Prep Kit for Illumina (NEB #7530, New England Biolabs, Ipswich, MA, USA). The purified double-stranded cDNA fragments underwent end repair, had an A base added, and were ligated to Illumina sequencing adapters. The products of ligation reaction were purified using the AMPure XP Beads (1.0X) and then amplified via polymerase chain reaction (PCR). The resulting cDNA library was sequenced on an Illumina Novaseq6000 platform by Gene Denovo Biotechnology Co. (Guangzhou, China).

**2.13 Data filtering and function annotation**

Reads obtained from the sequencing machines include raw reads containing adapters or low quality bases, which can impact subsequent assembly and analysis. Therefore, to obtain high-quality clean reads, the reads were further filtered using fastp (version 0.18.0). Differential expression analysis of RNAs was conducted between two different groups using DESeq2 software. Genes/transcripts with a false discovery rate (FDR) below 0.05 and an absolute fold change of≥2 were classified as differentially expressed genes (DEGs)/transcripts. Pathway enrichment analysis was carried out using the Kyoto Encyclopedia of Genes and Genomes (KEGG) database (<http://www.genome.jp/kegg>). Gene set enrichment analysis (GSEA) was conducted using GSEA software and MSigDB software to determine whether two groups exhibited significant differences in regard to a set of genes in specific KEGG pathways.

**2.14 GSH/GSSG assays**

The intracellular levels of GSH and GSSG were determined using a GSH and GSSG Assay Kit (S0053, Beyotime, Shanghai, China), in accordance with the manufacturer’s instructions. Briefly, cells were collected, lysed, and then successively subjected to –80˚C and 37˚C for rapid freezing and thawing. Subsequently, the cells were maintained at 4˚C for 5 min and centrifuged at 10,000×g for 10 min. The supernatant was combined with GSH assay buffer, GSH reductase, 5,5′-dithio-bis 2-nitrobenzoic acid solution, followed by incubation at 25˚C for 5 min. Subsequently, NADPH was added. Absorbance at 405 nm was determined using a microplate reader. The concentration of total glutathione, or GSSG, was determined from a standard curve, and GSH level was calculated as follows: GSH = (total glutathione−GSSG) × 2. The GSH/GSSG ratio was calculated as [GSH] / [GSSG].

**2.15 Labile iron pool (LIP) assays**

Excessive ferrous iron in the LIP leads to oxidative stress damage to cells through the Fenton reaction, which is a key step in driving ferroptosis. A549 and A549/DDP cells were collected at 48 hours post-cisplatin stimulation, with the cell count adjusted to 1×10^6^. The cells were labeled by adding 0.5 μM Calcein AM (C2012-0.1 ml, Beyotime, China) in PBS and incubated for 30 min at 37˚C. Following the incubation for 30 min, the cells were centrifuged at room temperature at a speed of 1500 rpm for 5 min. The collected cells were resuspended in 1 ml of PBS and then treated or untreated with 100 μM iron chelator DFO (D9533-1G, Sigma Aldrich, USA), followed by incubation at 37˚C in darkness for 1 hour. Fluorescence was determined using flow cytometry (BD Biosciences, San Jose, CA), at an excitation wavelength of 488 nm and an emission wavelength of 525 nm. The amount of LIP was inferred from the difference in mean fluorescence of each sample, with or without DFO treatment.

1. Okazaki K, Anzawa H, Liu Z, Ota N, Kitamura H, Onodera Y *et al*: **Enhancer remodeling promotes tumor-initiating activity in NRF2-activated non-small cell lung cancers**. *Nat Commun* 2020, **11**(1):5911.

2. Hanzelmann S, Castelo R, Guinney J: **GSVA: gene set variation analysis for microarray and RNA-seq data**. *BMC Bioinformatics* 2013, **14**:7.

3. Tai F, Zhai R, Ding K, Zhang Y, Yang H, Li H *et al*: **Long non‑coding RNA lung cancer‑associated transcript 1 regulates ferroptosis via microRNA‑34a‑5p‑mediated GTP cyclohydrolase 1 downregulation in lung cancer cells**. *Int J Oncol* 2024, **64**(6).
