# Supplementary material for: The Nrf2-HMOX1 pathway as a therapeutic target for reversing cisplatin resistance in non-small cell lung cancer via inhibiting ferroptosis
Source: Cell Death Discov. 2025 Jun 21;11:287. doi: 10.1038/s41420-025-02564-z (PMC12182566; doi:10.1038/s41420-025-02564-z)
Supplement: Supplementary file 2 — supplementary material for Fig S1 [file 41420_2025_2564_MOESM2_ESM.docx]

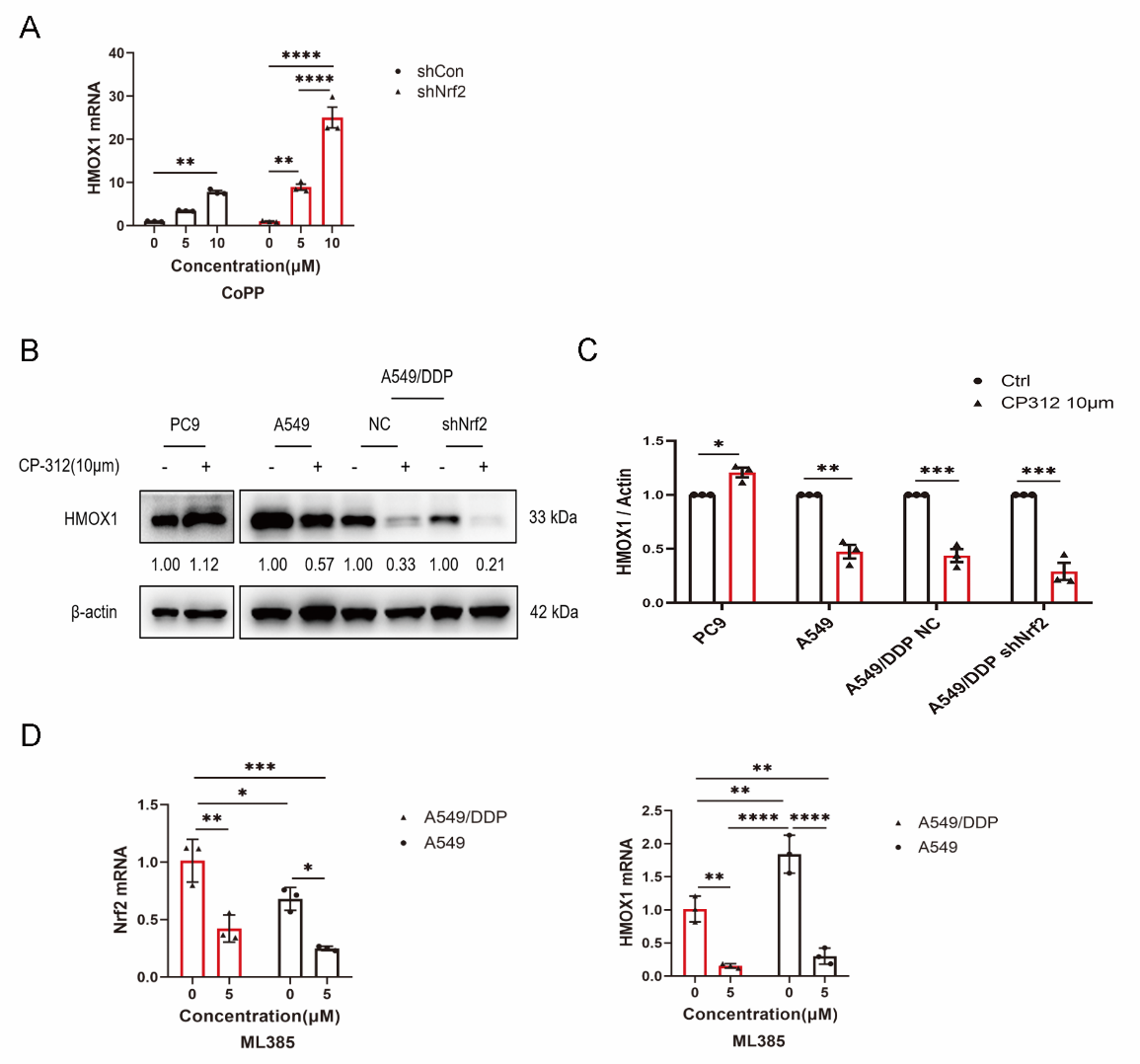


**FIG. S1** RT-PCR and WB results of gene expression following CoPP, CP-312 or ML385 treatments. (A) RT-PCR analysis of HMOX1 expression in both NC- and Nrf2-shRNA-infected A549/DDP cells following CoPP treatment. (B-C) PC9, A549, and A549/DDP cells transfected with negative control (NC) or Nrf2-shRNA were all treated with CP-312, an inducer of HMOX1, for 48 h. Western blot analysis assessed HMOX1 expression levels in all cell lines. The WB experiment was repeated three times with similar results. The grayscale values of the protein bands were quantified relative to the β-actin loading control via ImageJ software. (D) RT-PCR analysis of Nrf2 and HMOX1 expression in both A549 and A549/DDP cells following ML385 treatment. All statistical analysis was performed via unpaired t-tests, with **p*<0.05, ***p*<0.01, ****p*<0.001, and *****p*<0.0001 indicating significance relative to the control or differently treated groups.
