# Supplementary material for: The Nrf2-HMOX1 pathway as a therapeutic target for reversing cisplatin resistance in non-small cell lung cancer via inhibiting ferroptosis
Source: Cell Death Discov. 2025 Jun 21;11:287. doi: 10.1038/s41420-025-02564-z (PMC12182566; doi:10.1038/s41420-025-02564-z)
Supplement: Supplementary file 3 — Supplementary material for Table S1 [file 41420_2025_2564_MOESM3_ESM.docx]

| **Clinicopathological parameters** | **Total** | **Survival status** | | ***p* value** |
| --- | --- | --- | --- | --- |
|  |  | **Died（N=66)** | **Alive（N=27)** |  |
| **Gender** | | | | |
| Male | 57 | 41（73.21%） | 15（26.79%） | 0.557 |
| Female | 36 | 25（67.57%） | 12（32.43%） |  |
| **Age (years)** | | | | |
| <60 | 44 | 32（74.42%） | 11（25.58%） | 0.497 |
| >60 | 49 | 34（68.00%） | 16（32.00%） |  |
| **Smoking status** | | | | |
| Yes | 46 | 34（75.56%） | 11（24.44%） | 0.345 |
| No | 47 | 32（66.67%） | 16（33.33%） |  |
|  |  |  |  |  |
| **Tumor size (cm)** | | | | |
| <3 | 54 | 34（62.96%） | 20（37.04%） | 0.045^*^ |
| ≥3 | 39 | 32（82.05%） | 7（17.95%） |  |
| **Clinical stage（TNM）** | | | | |
| I +II | 64 | 32（57.14%） | 24（42.86%） | 0.0003^***^ |
| III +IV | 29 | 34（91.89%） | 3（8.11%） |  |
| **Pathology grade** | | | | |
| Poor | 21 | 24（96.00%） | 1（4.00%） |  |
| Moderate | 40 | 21（67.74%） | 10（32.26%） | 0.047^*^ |
| Well | 32 | 21（56.76%） | 16（43.24%） |  |
| **Lymph node metastasis** | | | | |
| No | 40 | 12（41.38%） | 17（58.62%） | <0.0001^***^ |
| Yes | 53 | 54（84.38%） | 10（15.63%） |  |
| **Distant metastasis status** | | | | |
| M0 | 81 | 45（63.38%） | 26（36.62%） | 0.004^**^ |
| M1 | 12 | 21（95.45%） | 1（4.55%） |  |
| **HMOX1 expression** | | | | |
| Low | 43 | 21（48.84%） | 22（51.16%） | <0.0001^***^ |
| High | 50 | 45（90.00%） | 5（10.00%） |  |
| Supplementary Table S1: Univariate analysis of clinicopathological parameters in NSCLC patients with respect to survival. Statistical analyses were performed by the Pearson χ2 test (*p<0.05, **p<0.01, ***p<0.001). | | | | |
