# Supplementary material for: The Nrf2-HMOX1 pathway as a therapeutic target for reversing cisplatin resistance in non-small cell lung cancer via inhibiting ferroptosis
Source: Cell Death Discov. 2025 Jun 21;11:287. doi: 10.1038/s41420-025-02564-z (PMC12182566; doi:10.1038/s41420-025-02564-z)
Supplement: Supplementary file 4 — Supplementary material for original western blots [file 41420_2025_2564_MOESM4_ESM.docx]

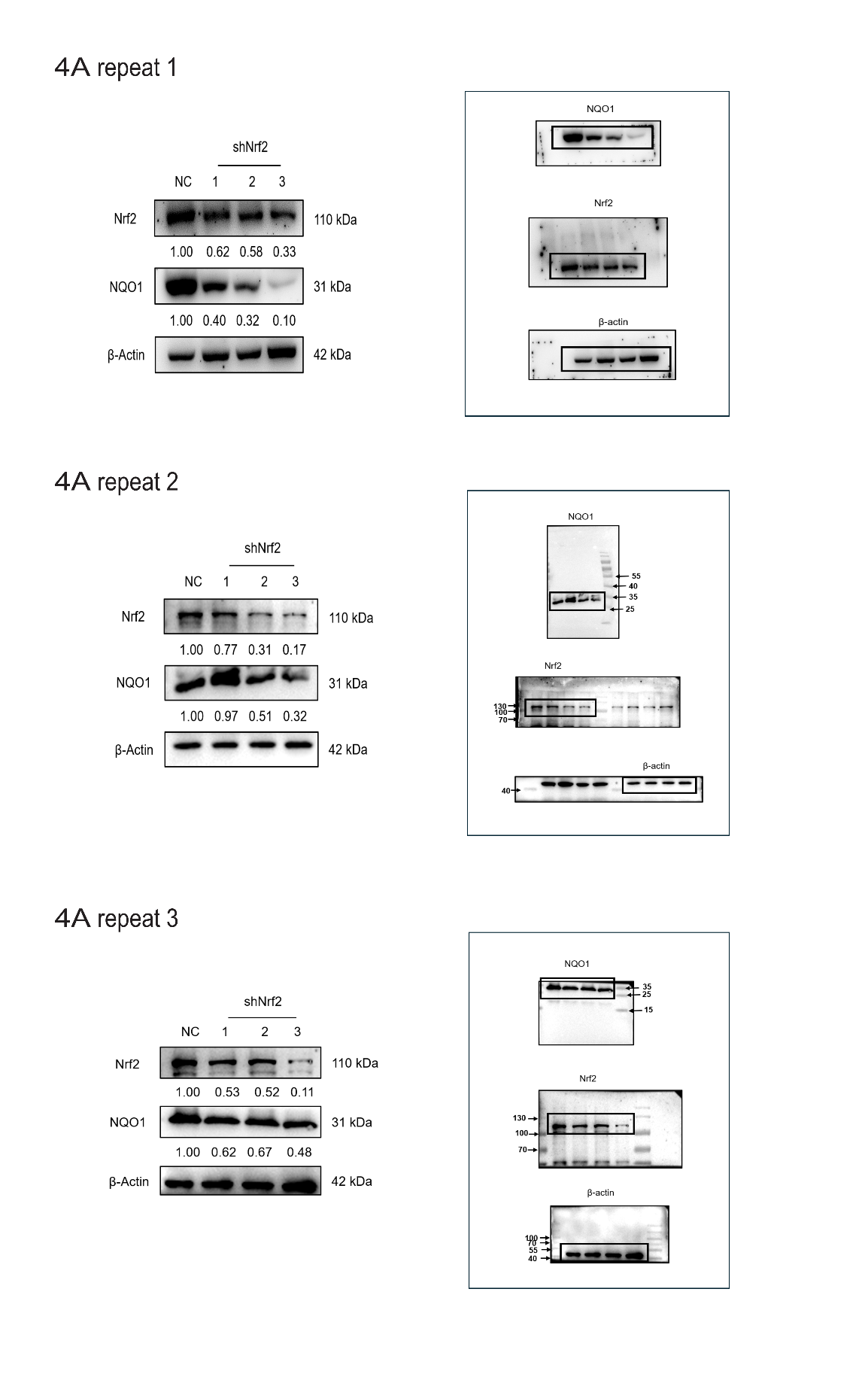


**FIG. 4A** Western blots analysis of Nrf2 and NQO1 expression levels in A549/DDP cells following Nrf2-shRNA infection. Three replicates are shown above. The original WB bands corresponding to each image are displayed on the right.


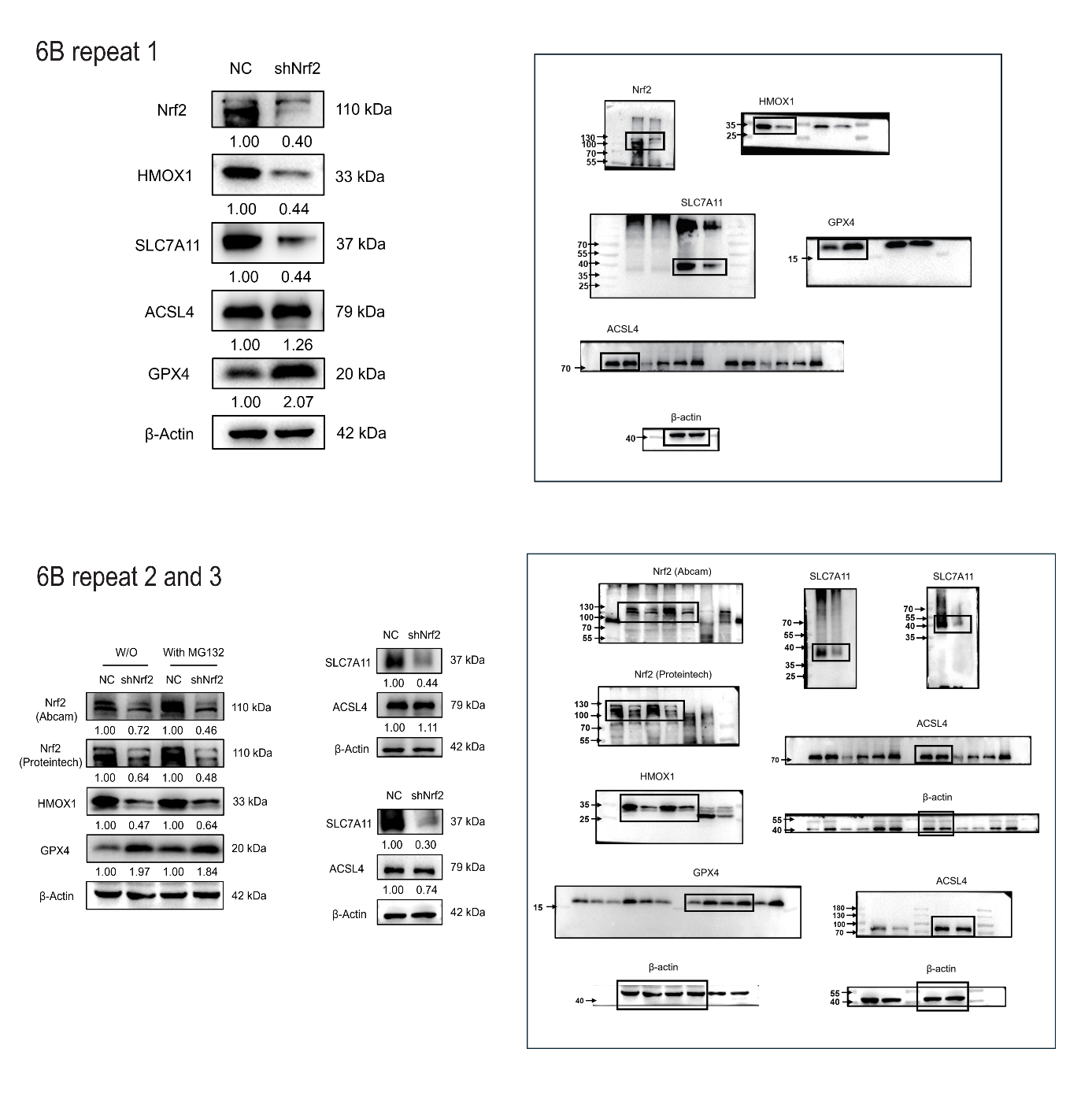


**FIG. 6B** Western blot analysis of Nrf2, HMOX1, SLC7A11, ACSL4, and GPX4 expression levels following Nrf2 knockdown in A549/DDP cells. Three replicates are shown above. In addition, in replicates 2 and 3, MG132 was added to reduce the ubiquitination degradation of Nrf2. The original WB bands corresponding to each image are displayed on the right.


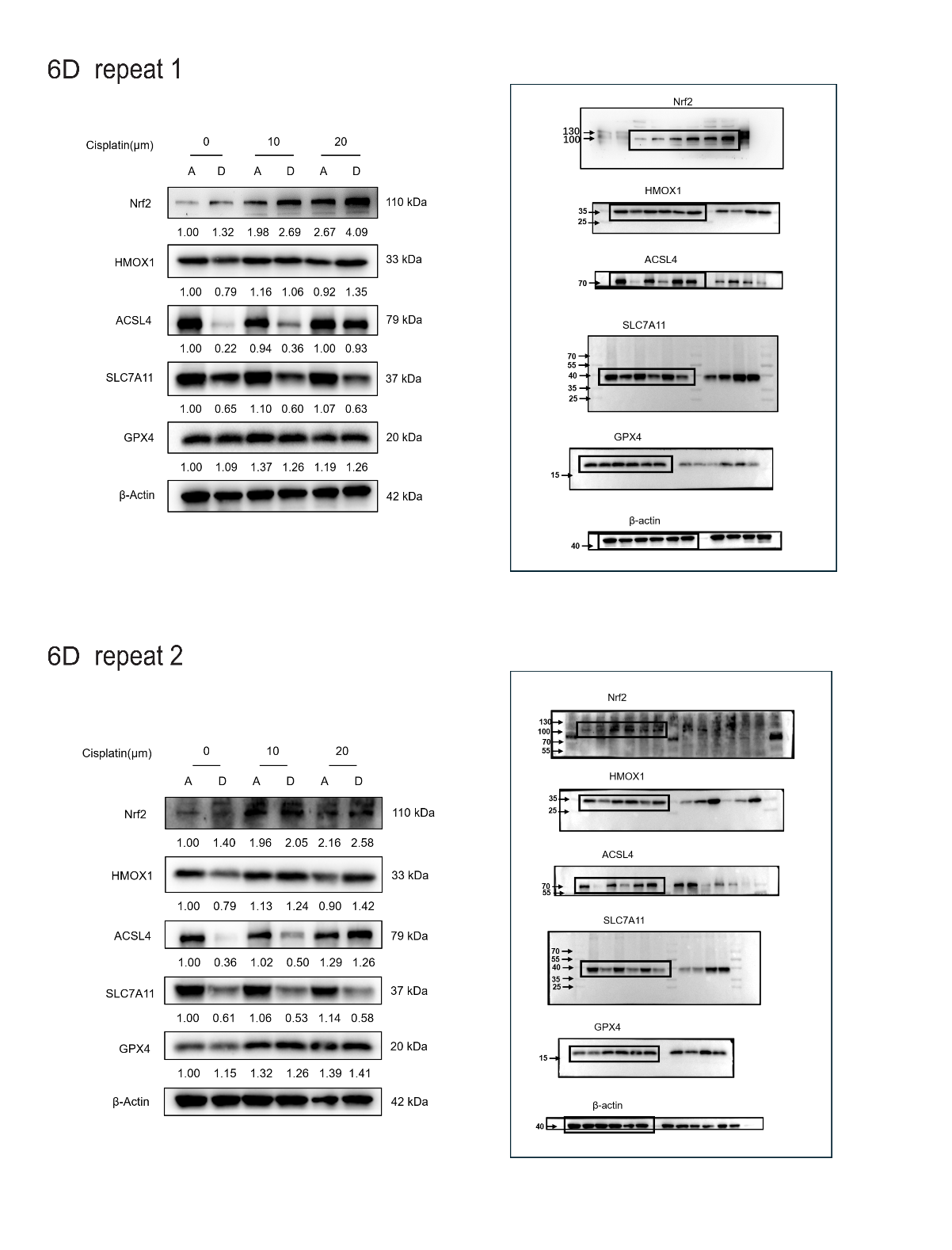


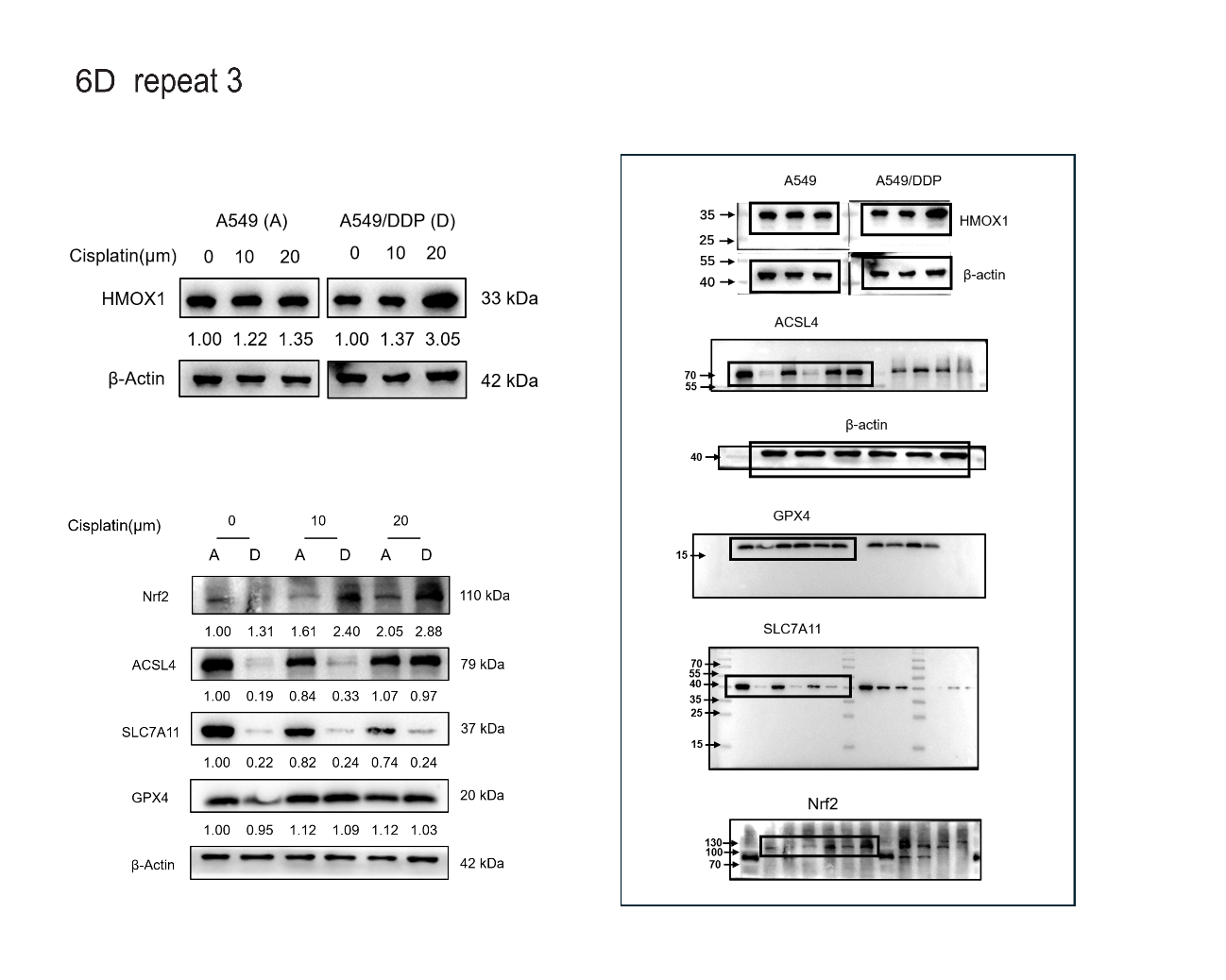


**FIG. 6D** Western blot analysis of Nrf2, HMOX1, ACSL4, SLC7A11, and GPX4 expression levels in A549 (A) and A549/DDP (D) cells treated with different concentrations of cisplatin for 48 hours. Three replicates are shown above. The original WB bands corresponding to each image are displayed on the right.


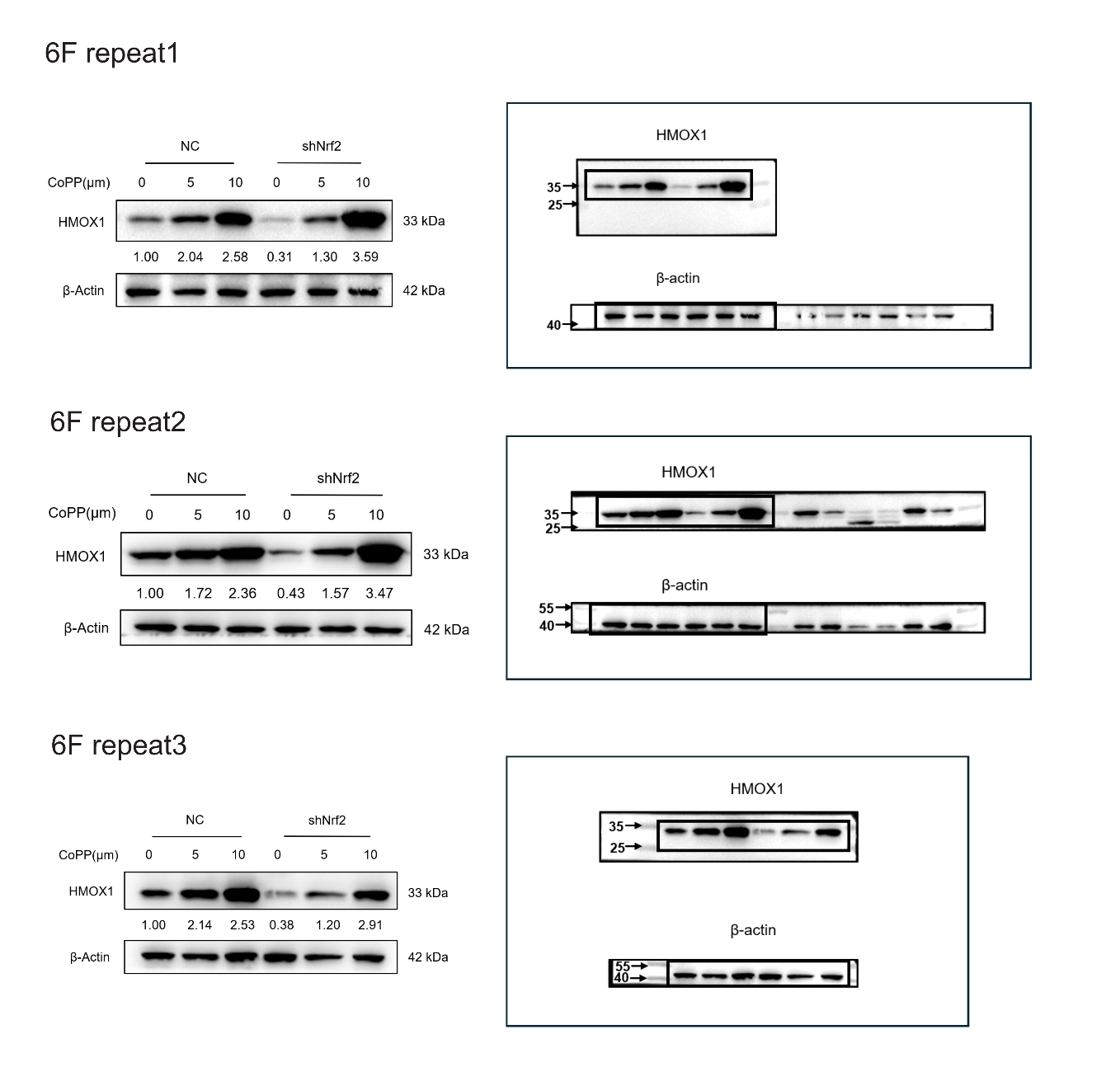


**FIG. 6F** Western blot analysis of HMOX1 expression levels in A549/DDP cells transfected with negative control (NC) or Nrf2-shRNA. Both cells were treated with CoPP, an inducer of HMOX1, for 48 h. Three replicates are shown above. The original WB bands corresponding to each image are displayed on the right.


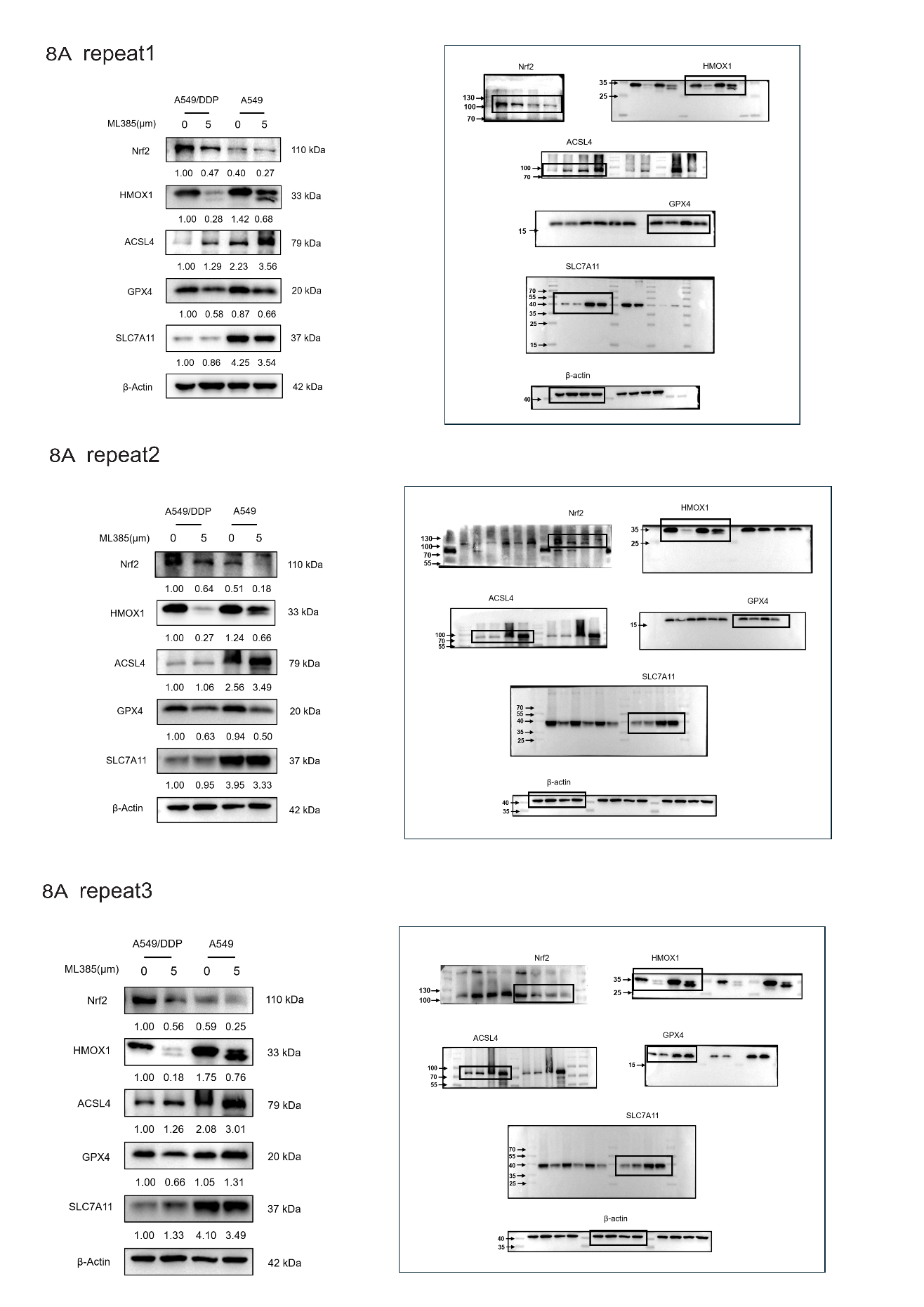


**FIG. 8A** Western blot analysis of Nrf2, HMOX1, ACSL4, GPX4, and SLC7A11 expression levels in both A549 and A549/DDP cells following treatment with the Nrf2 inhibitor ML385 (5 μM) for 48 h. Three replicates are shown above. The original WB bands corresponding to each image are displayed on the right.


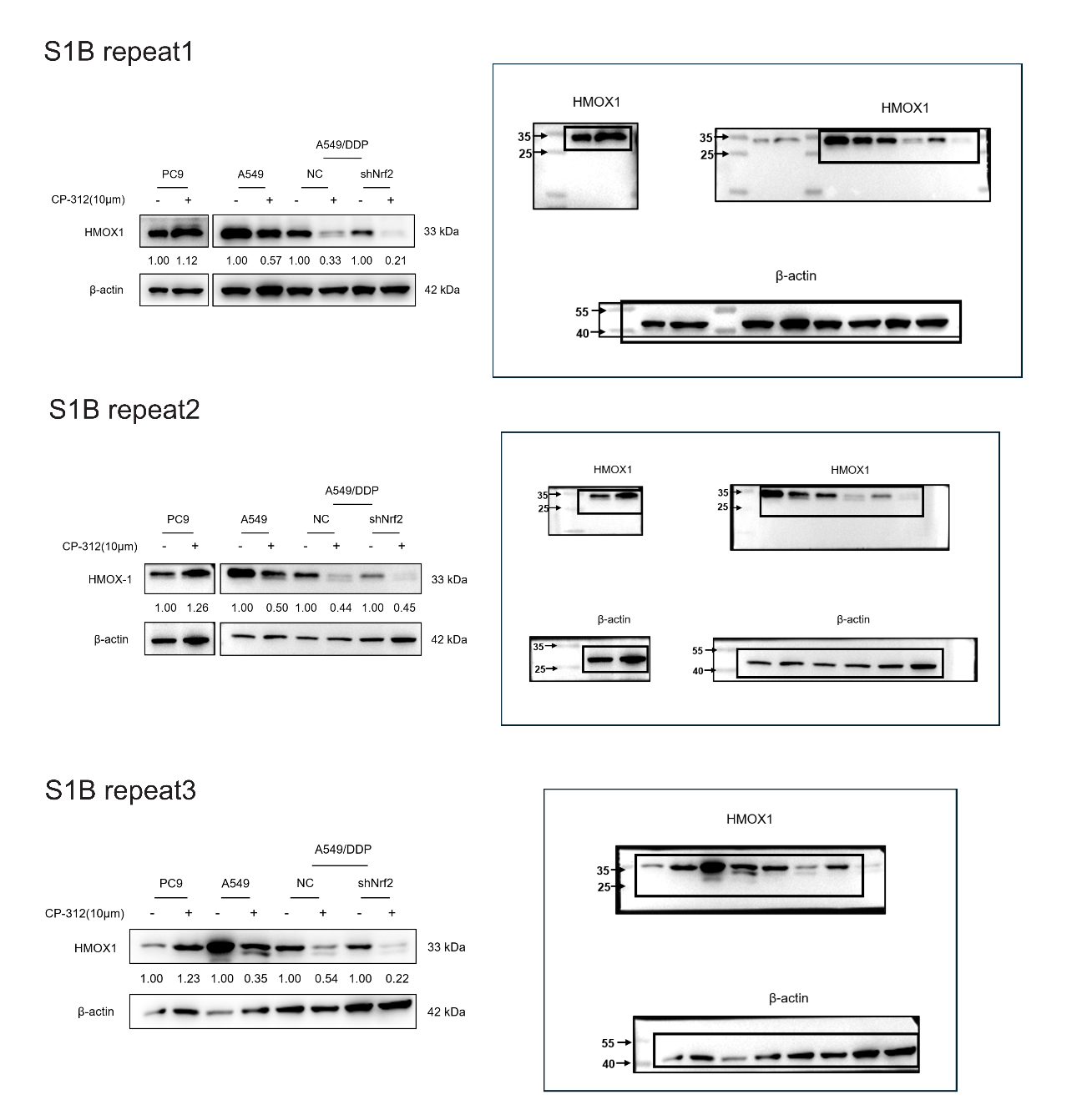


**FIG. S1B** Western blot analysis of HMOX1 expression levels in PC9, A549, and A549/DDP cells transfected with negative control (NC) or Nrf2-shRNA. All cell lines were treated with CP-312, another inducer of HMOX1, for 48 h. Three replicates are shown above. The original WB bands corresponding to each image are displayed on the right.
